# MISO Broadcast Channel under Unequal Link Coherence Times and Channel State Information

**DOI:** 10.3390/e22090976

**Published:** 2020-09-01

**Authors:** Mohamed Fadel Shady, Aria Nosratinia

**Affiliations:** Department of Electrical & Computer Engineering, The University of Texas at Dallas, Richardson, TX 75080, USA; mohamed.fadel@utdallas.edu

**Keywords:** broadcast channel, channel state information, coherence time, coherence diversity, degrees of freedom, fading channel, product superposition

## Abstract

The broadcast channel may experience unequal link coherence times due to a number of factors including variation in node mobility or local scattering conditions. This means the block fading model for different links may have nonidentical block length, and the channel state information for the links may also not be identical. The faster the fading and the shorter the fading block length, the more often the link needs to be trained and estimated at the receiver, and the more likely that channel state information (CSI) is stale or unavailable at the transmitter. This paper investigates a MISO broadcast channel where some receivers experience longer coherence intervals and other receivers experience shorter coherence intervals and must estimate their receive-side CSI (CSIR) frequently. We consider a variety of transmit-side CSI (CSIT) conditions for the abovementioned model, including no CSIT, delayed CSIT, or hybrid CSIT. To investigate the degrees of freedom region, we employ interference alignment and beamforming along with a product superposition that allows simultaneous but noncontaminating transmission of pilots and data to different receivers. Outer bounds employ the extremal entropy inequality as well as a bounding of the performance of a discrete, memoryless, multiuser, *multilevel* broadcast channel. For several cases, inner and outer bounds are established that either partially meet, or the gap diminishes with increasing coherence times.

## 1. Introduction

A typical wireless network is required to serve multiple users with different channel coherence and possibly also different quality of channel state information (CSI). For simplicity, most of the literature assumes similarity in CSI and channel coherence; with this assumption, the network capability and performance are limited by the users with the least CSI and channel coherence. In this paper, the assumption of uniformity in CSI and channel coherence is relaxed, allowing new gains in the network to be exploited.

The degrees of freedom (DoF) of a MIMO broadcast channel with similar CSI and channel coherence has been studied extensively. In the literature overview in this section, channels are single-input single-output (SISO) whenever no reference is made to the number of antennas. Under perfect instantaneous transmit-side CSI (CSIT) and receive-side CSI (CSIR), the degrees of freedom of a broadcast channel increase with the minimum of the transmit antennas and the total number of receive antennas [1,2]. Broadcast channel with perfect CSIR has been investigated under a variety of CSIT conditions, including imperfect, delayed, or no CSIT [3,4,5,6,7,8].

In the absence of CSIT, Huang et al. [3] and Vaze and Varanasi [4] showed that the degrees of freedom collapse to the single-user DoF, since the receivers are *stochastically equivalent* with respect to the transmitter. For a MISO broadcast channel, Lapidoth et al. [5] conjectured that as long as the precision of CSIT is finite, the degrees of freedom collapse to unity. This conjecture was recently settled in the positive by Davoodi and Jafar in [6]. Moreover, for a MISO broadcast channel under perfect delayed CSIT, Maddah-Ali and Tse in [7] showed using retrospective interference alignment that the degrees of freedom are $\frac{1}{1+\frac{1}{2}+\ldots +\frac{1}{K}}>1$, where *K* is the number of the transmit antennas and also the number of receivers. A scenario of mixed CSIT was investigated in [8], where the transmitter has partial knowledge about the current channel state in addition to delayed CSI.

The model of hybrid CSIT has been studied in the literature, where the CSIT with respect to different links may not be identical [6,9,10,11]. However, this model has assumed perfect and similar CSIR as well as identical coherence time for all users. A MISO broadcast channel with perfect CSIT for some receivers and delayed for the others was studied by Tandon et al. [9] and Amuru et al. [10]. Davoodi and Jafar [6] showed that for a MISO two-receiver broadcast channel under perfect CSIT for one user and no CSIT for the other, the degrees of freedom collapse to unity. Tandon et al. [11] considered a MISO broadcast channel with alternating hybrid CSIT to be perfect, delayed, or no CSIT with respect to different receivers.

With no CSIT for any users, the broadcast channel has been studied under unequal CSIR and unequal channel coherence time. An achievable degrees of freedom region for one slow-fading and one fast-fading receiver, the former with CSIR, was given in [12,13] via product superposition, discovering a gain that is now known as *coherence diversity*. Coherence diversity gain was further investigated in [14] for a *K*-receiver broadcast channel with neither CSIT nor CSIR.

In this paper, we consider a multiuser model in which a group of slow-fading receivers possessing longer block-fading are assumed to have CSIR; and another group of fast-fading receivers possessing shorter block-fading do not have CSIR a priori. We consider this model under a range of different CSIT conditions. The results of this paper are cataloged as follows.

*In the absence of CSIT,* an outer bound on the degrees of freedom region is produced via bounding the rates of a discrete, memoryless, multilevel broadcast channel [15,16] and then applying the extremal entropy inequality [17,18]. The outer bound is developed based on an extension to of the Körner–Marton outer bound ([19] Theorem 5) to more than two users. As a distinct contribution to this paper—the multiuser, discrete, memoryless, multilevel broadcast channel—we establish the capacity for degraded message sets, where one common message is communicated to all receivers and one further private message is communicated to one receiver.

*For delayed CSIT,* we use the outdated CSI model that was used by Maddah-Ali and Tse [7] under i.i.d. fading and assuming global CSIR at all nodes. Noting that our model does not have uniform CSIR, we produced a technique with alignment over super symbols to utilize outdated CSIT but merge it together with product superposition to reuse the pilots of the fast-fading receivers for the purpose of transmission to slow-fading receivers. Moreover, we develop an outer bound that is suitable for block-fading channels with different coherence times, by appropriately enhancing the channel to a physically-degraded broadcast channel and then applying the extremal entropy inequality [17,18]. For one slow-fading and one fast-fading receiver, our achievable degrees of freedom partially meet our outer bound, and furthermore, the gap decreases with the fast-fading receiver coherence time.

*Under hybrid CSIT,* we analyze two conditions: First, we consider perfect CSIT for the slow-fading receivers and no CSIT with respect to the fast-fading receivers. The achievable degrees of freedom in this case are obtained using product superposition with the fast-fading receiver’s pilots reused and beamforming for the slow-fading receivers to avoid interference. Second, we consider perfect CSIT with respect to the slow-fading receivers and delayed CSIT with respect to the fast-fading receivers. An achievable transmission scheme is proposed via a combination of beamforming, interference alignment, and product superposition methodologies. The outer bounds for the two hybrid-CSIT cases were based on constructing an enhanced physically degraded channel and then applying the extremal entropy inequality. For one slow-fading receiver with perfect CSIT and one fast-fading receiver with delayed CSIT, the gap between the achievable and the outer sum degrees of freedom is the inverse of the dynamic receiver coherence time.

## 2. System Model

**Figure 1:**
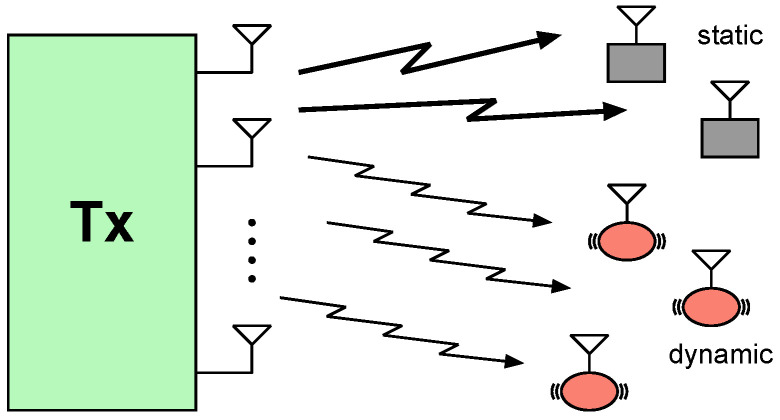
A broadcast channel with multiple slow-fading and multiple fast-fading users.

A taxonomy of the notation of this paper appears in Table 1. Consider a broadcast channel with multiple single-antenna receivers and the transmitter is equipped with $N_{t}$ antennas. The expressions “receiver” and “user” are employed without distinction throughout the paper, indicating the receiving terminals in the broadcast channel. The channels of the users are modeled as Rayleigh block-fading, where the channel coefficients remain constant over each block and change independently across blocks [20,21]. As shown in Figure 1, the users are partitioned into two sets based on channel availability and the length of the coherence interval: One set contains *m* fast-fading users with coherence time *T* and no CSIR, meaning that the cost of knowing CSI at the receiver—e.g., by channel estimation—is not ignored. The other set contains $m^{\prime }$ slow-fading users having coherence time $T^{\prime }$ and perfect instantaneous CSIR, where $T^{\prime }>>T$. We consider the transmitter is equipped with more antennas than the number of fast-fading and slow-fading users, i.e., $N_{t}\geq m^{\prime }+m$.

The received signals $y_{j}^{\prime }\left(t\right),y_{i}\left(t\right)$ at the slow-fading user *j* and the fast-fading user *i*, respectively, at time instant *t* are

(1) $$\begin{array}{cc} y_{j}^{\prime }\left(t\right) & =g_{j}^{†}\left(t\right)x\left(t\right)+z_{j}^{\prime }\left(t\right),\;j=1,\;\ldots ,\;m^{\prime },\\ y_{i}\left(t\right) & =h_{i}^{†}\left(t\right)x\left(t\right)+z_{i}\left(t\right),\;i=1,\;\ldots ,\;m, \end{array}$$

where $x\left(t\right)\in C^{N_{t}}$ is the transmitted signal, $z_{j}^{\prime }\left(t\right),z_{i}\left(t\right)$ denote the corresponding additive i.i.d. Gaussian noise of the users, and $g_{j}\left(t\right)\in C^{N_{t}},h_{i}\left(t\right)\in C^{N_{t}}$ denote the channels of the slow-fading user *j* and the fast-fading user *i* whose coefficients stay the same over $T^{\prime }$ and *T* time instances, respectively. The distributions of $g_{j}$ and $h_{i}$ are globally known at the transmitter and at the users (Additionally, the coherence times of all channels are globally known at the transmitter and at the users.). Having CSIR, the value of $g_{j}\left(t\right)$ is available instantaneously and perfectly at the slow-fading user *j*. Furthermore, the slow-fading user *j* obtains an outdated version of the fast-fading users’ channels $h_{i}$, and also the fast-fading user *i* obtains an outdated version of the slow-fading users’ channel $g_{i}$ (completely stale) [7]. CSIT for each user can take one of the following forms:

- Perfect CSIT: the channel vectors, $g_{j}\left(t\right),h_{i}\left(t\right)$, are available at the transmitter instantaneously and perfectly.
- Delayed CSIT: the channel vectors, $g_{j}\left(t\right),h_{i}\left(t\right)$, are available at the transmitter after they change independently in the following block (completely stale [7]).
- No CSIT: the channel vectors, $g_{j}\left(t\right),h_{i}\left(t\right)$, cannot be known at the transmitter.

We consider the broadcast channel with private messages for all users and no common messages. More specifically, we assume that the independent messages $M_{j}^{\prime }\in \left[1:2^{nR_{i}^{\prime }\left(\rho \right)}\right],M_{i}\in \left[1:2^{nR_{i}\left(\rho \right)}\right]$ associated with rates $R_{j}^{\prime }\left(\rho \right),R_{i}\left(\rho \right)$ are communicated from the transmitter to the slow-fading user *j* and fast-fading user *i*, respectively, at ρ signal-to-noise ratio. The degrees of freedom of the slow-fading and fast-fading users achieving rates $R_{j}^{\prime }\left(\rho \right),R_{i}\left(\rho \right)$ can be defined as

(2) $$\begin{array}{cc} d_{j}^{\prime } & =\lim_{\rho \to \infty }\frac{R_{j}^{\prime }\left(\rho \right)}{\log(\rho )},\;j=1,\;\ldots ,\;m^{\prime },\\ d_{i} & =\lim_{\rho \to \infty }\frac{R_{i}\left(\rho \right)}{\log(\rho )},\;i=1,\;\ldots ,\;m. \end{array}$$

The degrees of freedom region is defined as

(3) $$\begin{array}{cc} D=\{ & \left(d_{1}^{\prime },\;\ldots ,\;d_{m^{\prime }}^{\prime },d_{1},\;\ldots ,\;d_{m}\right)\in R_{+}^{m^{\prime }+m}|\;\exists \left(R_{1}^{\prime }\left(\rho \right),\;\ldots ,\;R_{m^{\prime }}^{\prime }\left(\rho \right),R_{1}\left(\rho \right),\;\ldots ,\;R_{m}\left(\rho \right)\right)\in C\left(\rho \right),\\ \; & d_{j}^{\prime }=\lim_{\rho \to \infty }\frac{R_{j}^{\prime }\left(\rho \right)}{\log(\rho )},d_{i}=\lim_{\rho \to \infty }\frac{R_{i}\left(\rho \right)}{\log(\rho )},\;j=1,\;\ldots ,\;m^{\prime },\;i=1,\;\ldots ,\;m\}, \end{array}$$

where C(ρ) is the capacity region at ρ signal-to-noise ratio. The sum degrees of freedom is defined as

(4) $$d_{\mathrm{sum}}=\lim_{\rho \to \infty }\frac{C_{\mathrm{sum}}\left(\rho \right)}{\log(\rho )},$$

where

(5) $$C_{\mathrm{sum}}\left(\rho \right)=\max\sum_{j=1}^{m^{\prime }}R_{j}^{\prime }\left(\rho \right)+\sum_{i=1}^{m}R_{i}\left(\rho \right).$$

In the sequel, we study the degrees of freedom of the above MISO broadcast channel under different CSIT scenarios that could be perfect, delayed, or no CSIT.

**Remark** **1.** *Under slow-fading, the degrees of freedom needed for channel training is a small fraction of the total degrees of freedom available in each fading block. The assumption of free CSIR essentially neglects this small overhead in the interest of simplicity. The authors in [14] studied the scenario of unequal coherence block length where no users are provided a priori CSIR. The extension of the results of this paper to two groups of users with two completely arbitrary fading-block lengths without free CSIR is possible via the methods of [14], but is not attempted herein in the interest of clarity and focus on the effect of different qualities and quantities of CSIT.*

## 3. No CSIT for Any Users

The broadcast channel defined in Section 2 is studied without CSIT. Bounding the rates of a multiuser, multilevel, discrete, memoryless broadcast channel in Section 3.1 provides the tools for outer bound on degrees of freedom of the channel of interest, in Section 3.2. Achievable degrees of freedom is obtained in Section 3.3.

### 3.1. Multiuser, Multilevel Broadcast Channel

**Figure 2:**
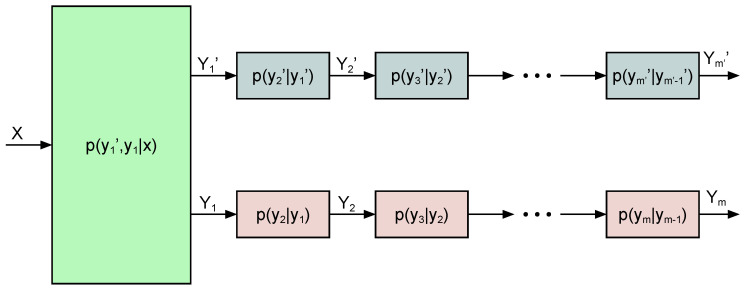
Discrete, memoryless, multiuser, multilevel broadcast channel.

The multilevel broadcast channel was introduced by Borade et al. [15] as a three-user broadcast, discrete, memoryless broadcast channel where two of the users are degraded with respect to each other. The capacity of this channel under degraded message sets was established by Nair and El Gamal [16]. Here, we study a *multiuser*, multilevel broadcast channel with two sets of degraded users (see Figure 2). One set contains $m^{\prime }$ users with $Y_{j}^{\prime }$ received signal at user *j*, and the other set contains *m* users with $Y_{i}$ received signal at user *i*. Therefore,

(6) $$\begin{array}{cc} X\to & Y_{1}^{\prime }\to Y_{2}^{\prime }\to \cdots \to Y_{m^{\prime }}^{\prime }\\ X\to & Y_{1}\to Y_{2}\to \cdots \to Y_{m} \end{array}$$

form two Markov chains. We consider a broadcast channel with $\left(m^{\prime }+m\right)$ private messages and no common message. An outer bound for the above multilevel broadcast channel is given in the following theorem.

**Theorem** **1.** *The rate region of the multilevel broadcast channel with two sets of degraded users (Equation (6)) is outer bounded by the intersection of*

(7) $$\begin{array}{cc} R_{1}\leq & I\left(U_{m^{\prime }},W;Y_{1}|V_{1}\right)-I\left(W;Y_{m^{\prime }}^{\prime }|U_{m^{\prime }}\right), \end{array}$$

(8) $$\begin{array}{cc} R_{i}\leq & I(V_{i-1};Y_{i}|V_{i}),\;\;i=2,\;\ldots ,\;m, \end{array}$$

(9) $$\begin{array}{cc} R_{j}^{\prime }\leq & I\left(U_{j-1};Y_{j}^{\prime }|U_{j}\right),\;\;j=1,\;\ldots ,\;m^{\prime }-1, \end{array}$$

(10) $$\begin{array}{cc} R_{m^{\prime }}^{\prime }\leq & I\left(W;Y_{m^{\prime }}^{\prime }|U_{m^{\prime }}\right)+I\left(X;Y_{m^{\prime }}^{\prime }|U_{m^{\prime }},W\right)-I\left(X;Y_{m^{\prime }}^{\prime }|U_{m^{\prime }-1}\right), \end{array}$$

*and*

(11) $$\begin{array}{cc} R_{i}\leq & I({\tilde{U}}_{i-1};Y_{i}|{\tilde{U}}_{i}),\;\;i=1,\;\ldots ,\;m-1, \end{array}$$

(12) $$\begin{array}{cc} R_{m}\leq & I\left(\tilde{W};Y_{m}|{\tilde{U}}_{m}\right)+I\left(X;Y_{m}|{\tilde{U}}_{m},\tilde{W}\right)-I\left(X;Y_{m}|{\tilde{U}}_{m-1}\right), \end{array}$$

(13) $$\begin{array}{cc} R_{1}^{\prime }\leq & I\left({\tilde{U}}_{m},\tilde{W};Y_{1}^{\prime }|{\tilde{V}}_{1}\right)-I\left(\tilde{W};Y_{m}|{\tilde{U}}_{m}\right), \end{array}$$

(14) $$\begin{array}{cc} R_{j}^{\prime }\leq & I\left({\tilde{V}}_{j-1};Y_{j}^{\prime }|{\tilde{V}}_{j}\right),\;\;j=2,\;\ldots ,\;m^{\prime }, \end{array}$$

*for some pmf*

(15) $$p(u_{1},\;\ldots ,u_{m^{\prime }},{\tilde{u}}_{1},\;\ldots ,\;{\tilde{u}}_{m},v_{1},\;\ldots ,v_{m},{\tilde{v}}_{1},\;\ldots ,\;{\tilde{v}}_{m^{\prime }},w,\tilde{w},x),$$

*where*

(16) $$\begin{array}{cc} U_{m^{\prime }}\to \cdots \to U_{1}\to & X\to (Y_{1},\;\ldots ,\;Y_{m},Y_{1}^{\prime },\;\ldots ,\;Y_{m^{\prime }}^{\prime })\\ V_{m}\to \cdots \to V_{1}\to \left(W,U_{m^{\prime }}\right)\to & X\to (Y_{1},\;\ldots ,\;Y_{m},Y_{1}^{\prime },\;\ldots Y_{m^{\prime }}^{\prime })\\ {\tilde{U}}_{m}\to \cdots \to {\tilde{U}}_{1}\to & X\to (Y_{1},\;\ldots Y_{m},Y_{1}^{\prime },\;\ldots ,\;Y_{m^{\prime }}^{\prime })\\ {\tilde{V}}_{m^{\prime }}\to \cdots \to {\tilde{V}}_{1}\to \left(\tilde{W},{\tilde{U}}_{m}\right)\to & X\to (Y_{1},\;\ldots Y_{m},Y_{1}^{\prime },\;\ldots ,\;Y_{m^{\prime }}^{\prime }) \end{array}$$

*forms Markov chains and $U_{0}={\tilde{U}}_{0}≜X$.*

**Proof.**  See Appendix A. □

**Remark** **2.** *Theorem 1 is an extension of the Körner–Marton outer bound ([19] Theorem 5) to more than two users, and it recovers the Körner–Marton bound when $m=m^{\prime }=1$.*

**Remark** **3.** *For the multiuser, multilevel broadcast channel characterized by (6), we establish the capacity for degraded message sets in Appendix B, where one common message is communicated to all receivers and one further private message is communicated to one receiver.*

### 3.2. Outer Degrees of Freedom Region

We now return to the broadcast channel defined in Section 2.

**Theorem** **2.** *An outer bound on the degrees of freedom region of the fading broadcast channel characterized by Equation (1), without CSIT, is*

(17) $$\begin{array}{cc} \sum_{j=1}^{m^{\prime }}d_{j}^{\prime } & \leq 1, \end{array}$$

(18) $$\begin{array}{cc} \sum_{i=1}^{m}d_{i} & \leq 1-\frac{1}{T}, \end{array}$$

(19) $$\begin{array}{cc} \sum_{j=1}^{m^{\prime }}d_{j}^{\prime }+\sum_{i=1}^{m}d_{i} & \leq \frac{4}{3}. \end{array}$$

**Proof.**  Equations (17) and (18) are, respectively, outer bounds for the slow-fading users alone and fast-fading users alone. These are bounds on the sum-DoF of a broadcast channel whose receivers have the same fading-block length [14,22]. The remainder of the proof is dedicated to establishing (19). We enhance the channel by giving all users global CSIR. Having no CSIT, the channel belongs to the class of multiuser, multilevel broadcast channels in Section 3.1. We then use the two outer bounds developed for the multilevel broadcast channels to generate two degrees of freedom bounds, and merge them to get the desired result.

We begin with the outer bound described in (7)–(10); we combine these equations to obtain partial sum-rate bounds on the slow-fading $\left(\sum R_{j}^{\prime }\right)$ and fast-fading $\left(\sum R_{i}\right)$ receivers:

(20) $$\begin{array}{cc} \sum_{j=1}^{m^{\prime }}R_{j}^{\prime }\leq & \sum_{j=1}^{m^{\prime }-1}I\left(U_{j-1};y_{j}^{\prime }|U_{j},H\right)+I\left(W;y_{m^{\prime }}^{\prime }|U_{m^{\prime }},H\right)+I\left(x;y_{m^{\prime }}^{\prime }|U_{m^{\prime }},W,H\right)\\ \; & -I(x;y_{m^{\prime }}^{\prime }|U_{m^{\prime }-1},H)\\ = & \sum_{j=1}^{m^{\prime }-1}h\left(y_{j}^{\prime }|U_{j},H\right)-h\left(y_{j}^{\prime }|U_{j-1},H\right)+I\left(W;y_{m^{\prime }}^{\prime }|U_{m^{\prime }},H\right)+h\left(y_{m^{\prime }}^{\prime }|U_{m^{\prime }},W,H\right)\\ \; & -h\left(y_{m^{\prime }}^{\prime }|U_{m^{\prime }-1},H\right)+o\left(\log\left(\rho \right)\right) \end{array}$$

(21) $$\begin{array}{cc} = & I\left(W;y_{m^{\prime }}^{\prime }|U_{m^{\prime }},H\right)+h\left(y_{m^{\prime }}^{\prime }|U_{m^{\prime }},W,H\right)+o\left(\log\left(\rho \right)\right), \end{array}$$

where H is the set of all channel vectors; (20) follows from the chain rule, $h\left(y_{j}^{\prime }|x,H\right)=o\left(\log\left(\rho \right)\right)$; and (21) follows since the received signals of all slow-fading users, $y_{j}^{\prime }$, have the same statistics [14,22]. Additionally, using Theorem 1,

(22) $$\begin{array}{cc} \sum_{j=1}^{m}R_{j}\leq & I\left(U_{m^{\prime }},W;y_{1}|V_{1},H\right)-I\left(W;y_{m^{\prime }}^{\prime }|U_{m^{\prime }},H\right)+\sum_{j=2}^{m}I\left(V_{j-1};y_{j}|V_{j},H\right)\\ = & h\left(y_{1}|V_{1},H\right)-h\left(y_{1}|U_{m^{\prime }},W,H\right)-I\left(W;y_{m^{\prime }}^{\prime }|U_{m^{\prime }},H\right)+\sum_{j=2}^{m}h\left(y_{j}|V_{j},H\right)\\ \; & -h(y_{j}|V_{j-1},H) \end{array}$$

(23) $$\begin{array}{cc} = & -h\left(y_{1}|U_{m^{\prime }},W,H\right)-I\left(W;y_{m^{\prime }}^{\prime }|U_{m^{\prime }},H\right)+h\left(y_{m}|V_{m},H\right)+o\left(\log\left(\rho \right)\right) \end{array}$$

(24) $$\begin{array}{cc} \leq & -h\left(y_{1}|U_{m^{\prime }},W,H\right)-I\left(W;y_{m^{\prime }}^{\prime }|U_{m^{\prime }},H\right)+\log\left(\rho \right)+o\left(\log\left(\rho \right)\right), \end{array}$$

where (22) follows from the chain rule, (23) follows since $y_{j}$ have the same statistics, and (24) follows since $h\left(y_{m}|V_{m},H\right)\leq n\log\left(\rho \right)+o\left(\log\left(\rho \right)\right)$. Define $Y_{j,k}^{\prime }$ to be the received signal of user *j* at time instance *k*. From (21) and (24), we can obtain the bound (27) on the rates.

(25) $$\begin{array}{cc} \frac{1}{2}\sum_{j=1}^{m^{\prime }}R_{j}^{\prime }+\sum_{j=1}^{m}R_{j}\leq & \frac{1}{2}I\left(W;y_{m^{\prime }}^{\prime }|U_{m^{\prime }},H\right)+\frac{1}{2}h\left(y_{m^{\prime }}^{\prime }|U_{m^{\prime }},W,H\right)-h\left(y_{1}|U_{m^{\prime }},W,H\right)\\ \; & -I\left(W;y_{m^{\prime }}^{\prime }|U_{m^{\prime }},H\right)+\log\left(\rho \right)+o\left(\log\left(\rho \right)\right)\\ = & \frac{1}{2}h\left(y_{m^{\prime }}^{\prime }|U_{m^{\prime }},W,H\right)-h\left(y_{1}|U_{m^{\prime }},W,H\right)+\log\left(\rho \right)+o\left(\log\left(\rho \right)\right)\\ \leq & \frac{1}{2}h\left(y_{m^{\prime }}^{\prime },y_{1}|U_{m^{\prime }},W,H\right)-h\left(y_{1}|U_{m^{\prime }},W,H\right)+\log\left(\rho \right)+o\left(\log\left(\rho \right)\right) \end{array}$$

(26) $$\begin{array}{cc} \leq & \sum_{k=1}^{n}\frac{1}{2}h\left(y_{m^{\prime },k}^{\prime },y_{1,k}|U_{m^{\prime }},W,H,y_{m^{\prime },1}^{\prime },\;\ldots ,\;y_{m^{\prime },k-1}^{\prime },y_{1,1},\;\ldots ,\;y_{1,k-1}\right)\\ \; & -h\left(y_{1,k}|U_{m^{\prime }},W,H,y_{m^{\prime },1}^{\prime },\;\ldots ,\;y_{m^{\prime },k-1}^{\prime },y_{1,1},\;\ldots ,\;y_{1,k-1}\right)+\log\left(\rho \right)\\ \; & +o(\log(\rho )) \end{array}$$

(27) $$\begin{array}{cc} \leq & \max_{\mathrm{Tr}\left\{\Sigma_{x}\right\}\leq \rho ,\Sigma_{x}≽0}E_{H}\left\{\frac{1}{2}\log\left|I+H\Sigma_{x}H^{†}\right|-\log\left(1+h_{1}^{†}\Sigma_{x}h_{1}\right)\right\}+\log\left(\rho \right)\\ \; & +o(\log(\rho )), \end{array}$$

where (25) and (26) follow from the chain rule that conditioning does not increase differential entropy, and (27) follows from extremal entropy inequality [17,18,23]. In order to bound (27), we use a specialization of [24] Lemma 3 as follows.

**Lemma** **1.** *Consider two random matrices $H_{1}\in C^{N_{1}\times N_{t}}$ and $H_{2}\in C^{N_{2}\times N_{t}}$, where $N_{1}\geq N_{2}$. For a covariance matrix $\Sigma_{x}$, where Tr$\{\Sigma_{x}\}\leq \rho$, we have*

(28) $$\begin{array}{cc} \max_{\Sigma_{x}}\; & \frac{1}{\min\{N_{t},N_{1}\}}\log|I+H_{1}\Sigma_{x}H_{1}^{†}|-\frac{1}{\min\{N_{t},N_{2}\}}\log\left|I+H_{2}\Sigma_{x}H_{2}^{†}\right|\leq o\left(\log\left(\rho \right)\right). \end{array}$$

The proof of Lemma 1 is omitted as it directly follows from [24] Lemma 3. Lemma 1 yields the following outer bound on the degrees of freedom:

(29) $$\frac{1}{2}\sum_{j=1}^{m^{\prime }}d_{j}^{\prime }+\sum_{i=1}^{m}d_{i}\leq 1.$$

We now repeat the exercise of bounding the sum rates and deriving degrees of freedom, this time starting from (11)–(14). By following bounding steps parallel to (21), (24), and (27),

(30) $$\sum_{j=1}^{m^{\prime }}d_{j}^{\prime }+\frac{1}{2}\sum_{i=1}^{m}d_{i}\leq 1.$$

Adding (29) and (30) yields the outer bound (19), completing the proof of Theorem 2. □

### 3.3. Achievable Degrees of Freedom Region

**Theorem** **3.** *The fading broadcast channel described by Equation (1) can achieve the following degrees of freedom without CSIT:*

(31) $$\begin{array}{cc} \sum_{i=1}^{m}d_{i} & \leq 1-\frac{1}{T}, \end{array}$$

(32) $$\begin{array}{cc} \sum_{j=1}^{m^{\prime }}d_{j}^{\prime }+\sum_{i=1}^{m}d_{i} & \leq 1. \end{array}$$

**Proof.**  The achievable scheme uses product superposition [13,22], where the transmitter uses one antenna to send the super symbol to two users: one fast-fading and one slow-fading,

(33) $$x^{†}=x_{s}x_{d}^{†},$$

where $x_{s}\in C$ is a symbol intended for the slow-fading user; and

(34) $$x_{d}^{†}=\left[x_{\tau },\;\;x_{\delta }^{†}\right],$$

where $x_{\tau }\in C$ is a pilot and $x_{\delta }\in C^{T-1}$ is a super symbol intended for the fast-fading user. Since degrees of freedom analysis is insensitive to the additive noise, we omit the noise component in the following.

(35) $$\begin{array}{cc} y^{†} & =hx_{s}\left[x_{\tau },\;\;x_{\delta }^{†}\right]\\ \; & =[\bar{h}x_{\tau },\;\;\bar{h}x_{\delta }^{†}], \end{array}$$

where $\bar{h}=hx_{s}$. The fast-fading user estimates the equivalent channel $\bar{h}$ during the first time instance and then decodes $x_{\delta }$ *coherently* based on the channel estimate. The slow-fading receiver only utilizes the received signal during the first time instance:

(36) $$y_{1}^{\prime }=gx_{s}.$$

Knowing its channel gain *g*, the slow-fading receiver can decode $x_{s}$. The achievable degrees of freedom of the two users are

(37) $$\left(d^{\prime },d\right)=\left(\frac{1}{T},1-\frac{1}{T}\right).$$

**Figure 3:**
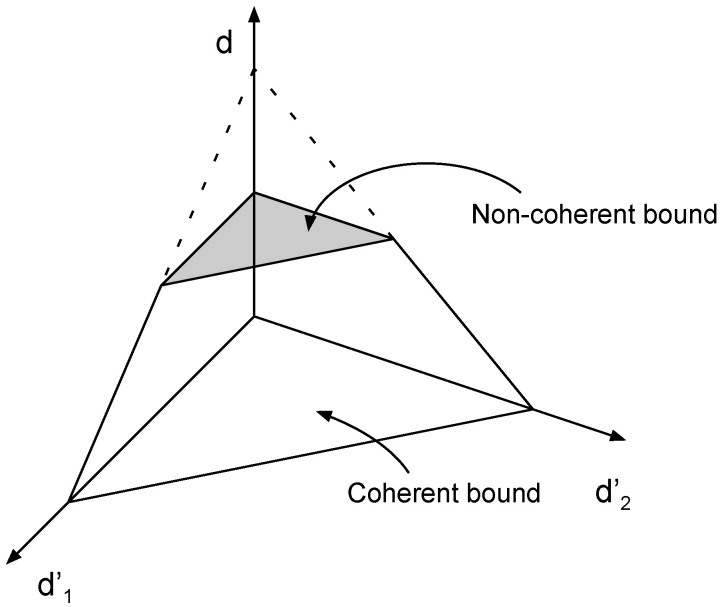
Achievable degrees of freedom region of one fast-fading and two slow-fading users.

**Figure 4:**
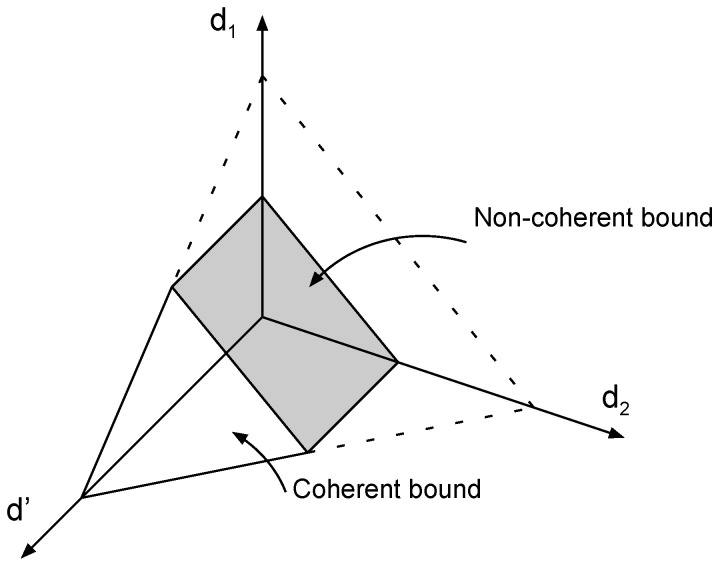
Achievable degrees of freedom region of one slow-fading and two fast-fading users.

We now proceed to prove that the degrees of freedom region characterized by (31) and (32) can be achieved via a combination of two-user product superposition strategies that were outlined above, and single-user strategies. For clarity of exposition we refer to (31)—which describes the degrees of freedom constraints of the fast-fading receivers—as the *noncoherent bound*, and to (32) as the *coherent bound*. The non-negativity of degrees of freedom restricts them to the non-negative orthant $R_{+}^{m+m^{\prime }}$. The intersection of the coherent bound and the non-negative orthant is a $\left(m^{\prime }+m\right)$–simplex that has $m+m^{\prime }+1$ vertices. The noncoherent bound is a hyperplane that partitions the simplex with $m^{\prime }+1$ vertices on one side of the noncoherent bound and *m* on the other. Therefore, the intersection of the simplex with the noncoherent bound produces a polytope with $\left(m^{\prime }+1\right)\left(m+1\right)$ vertices (This can be verified with a simple counting exercise involving the number of edges of the simplex that cross the noncoherent bound.). For illustration, see Figure 3 showing the three-user degrees of freedom with two slow-fading users and Figure 4 with one slow-fading user.

We now verify that each of the $\left(m^{\prime }+1\right)\left(m+1\right)$ vertices can be achieved with either a single-user strategy, or via a two-user product superposition strategy:

- $m^{\prime }$ vertices corresponding to single-user transmission to each slow-fading user *j* achieving one degree of freedom.
- *m* vertices corresponding to single-user transmission to each fast-fading user *i* achieving $\left(1-\frac{1}{T}\right)$ degrees of freedom.
- $m^{\prime }m$ vertices corresponding to product superposition applied to all possible pairs of slow-fading and fast-fading users, achieving $\frac{1}{T}$ degrees of freedom for one slow-fading user and $\left(1-\frac{1}{T}\right)$ degrees of freedom for one fast-fading user.
- One trivial vertex at the origin, corresponding to no transmission, achieving zero degrees of freedom for all users.

Hence, the number of the vertices is $m^{\prime }+m+m^{\prime }m+1=\left(m+1\right)\left(m^{\prime }+1\right)$. This completes the achievability Proof of Theorem 3. □

## 4. Delayed CSIT for All Users

Under delayed CSIT, the transmitter knows each channel gain only after it is no longer valid. This condition is also known as outdated CSIT. We begin by proving inner and outer bounds when transmitting only to slow-fading users, only to fast-fading users, and to one slow-fading and one fast-fading user. We then synthesize this collection of bounds into an overall degrees of freedom region.

### 4.1. Transmission to Slow-Fading  Users

**Theorem** **4.** *The degrees of freedom region of the fading broadcast channel characterized by Equation (1), with delayed CSIT and having $m^{\prime }$ slow-fading users and no fast-fading users is*

(38) $$d_{j}^{\prime }\leq \frac{1}{1+\frac{1}{2}+\ldots +\frac{1}{m^{\prime }}},\;j=1,\;\ldots ,\;\;m^{\prime }.$$

**Proof.**  The case of $T^{\prime }=1$ was discussed by Maddah-Ali and Tse in [7], where the achievability was established by *retrospective interference alignment* that aligns the interference using the outdated CSIT; and the converse was proved by generating an improved channel without CSIT having a tight degrees of freedom region against TDMA according to the results in [3,4]. For $T^{\prime }\geq 1$, the achievability is established by employing retrospective interference alignment presented in [7] over super symbols, each of length $T^{\prime }$. The converse is proved by following the same procedures in [7] to generate a block-fading improved channel without CSIT and with identical coherence intervals of length $T^{\prime }$. According to the results of [14,22], TDMA is tight against the degrees of freedom region of the improved channel. □

### 4.2. Transmission to Fast-Fading Users

**Theorem** **5.** *The fading broadcast channel characterized by Equation (1), with delayed CSIT and having m fast-fading users and no slow-fading users, can achieve the degrees of freedom*

(39) $$d_{i}\leq \frac{1}{1+\frac{1}{2}+\ldots +\frac{1}{m}}\left(1-\frac{m}{T}\right),\;i=1,\;\ldots ,\;m.$$

*An outer bound on the degrees of freedom region is*

(40) $$\begin{array}{cc} d_{i}\leq & 1-\frac{1}{T}, \end{array}$$

(41) $$\begin{array}{cc} \sum_{i=1}^{m}d_{i}\leq & \frac{m}{1+\frac{1}{2}+\ldots +\frac{1}{m}}. \end{array}$$

**Proof.**  The achievability part can be proved as follows. At the beginning of each super symbol, *m* pilots are sent for channel estimation. Then, retrospective interference alignment in [7] over super symbols is employed during the remaining (T−m) instances to achieve (39). For the converse part, (41) is proved by giving the users global CSIR, and then applying Theorem 4. Moreover, (40) is the single-user bound for each fast-fading user that can be proved as follows. For a single user with delayed CSIT, feedback does not increase the capacity [25]; consequently, the assumption of delayed CSIT can be removed. Hence, the single-user bound for each fast-fading user with delayed CSIT is the same as the single-user bound without CSIT [21]. □

### 4.3. Transmission to One Slow-Fading and One Fast-Fading User

**Theorem** **6.** *The fading broadcast channel characterized by Equation (1), with delayed CSIT and having one slow-fading and one fast-fading user, can achieve the following degrees of freedom*

(42) $$\begin{array}{cc} D_{1} & :\left(d^{\prime },d\right)=\left(\frac{2}{3}\left(1+\frac{1}{T}\right),\frac{2}{3}\left(1-\frac{2}{T}\right)\right), \end{array}$$

(43) $$\begin{array}{cc} D_{2} & :\left(d^{\prime },d\right)=\left(\frac{1}{T},1-\frac{1}{T}\right). \end{array}$$

*Furthermore, the achievable degrees of freedom region is the convex hull of the above degrees of freedom pairs.*

**Proof.**  From Section 3.3, product superposition achieves the pair (43) that does not require CSIT for any of the two users. The remainder of the proof is dedicated to the achievability of the pair (42). We provide a transmission scheme based on retrospective interference alignment [7] along with product superposition.

1. The transmitter first emits a super symbol intended for the slow-fading user:
  (44) $$X_{1}=\left[X_{1,1},\;\;\cdots ,\;\;X_{1,\ell }\right],$$
  where $\ell =\frac{T^{\prime }}{T}$, and each $X_{1,n}\in C^{2\times T}$ occupies *T* time instances and has the following structure:
  (45) $$X_{1,n}=\left[{\bar{U}}_{n},\;\;{\bar{U}}_{n}U_{n}\right],\;n=1,\;\ldots ,\;\ell ,$$
  both the diagonal matrix ${\bar{U}}_{n}\in C^{2\times 2}$ and $U_{n}\in C^{2\times (T-2)}$ contain symbols intended for the slow-fading user. The components of $y_{1}^{{}^{\prime }†}=\left[y_{1,1}^{{}^{\prime }†},\;\;\cdots ,\;\;y_{1,\ell }^{{}^{\prime }†}\right]$ are
  (46) $$\begin{array}{cc} y_{1,n}^{{}^{\prime }†}= & [g_{1}^{†}{\bar{U}}_{n},\;\;g_{1}^{†}{\bar{U}}_{n}U_{n}],\;n=1,\;\ldots ,\;\ell \\ = & [{\tilde{g}}_{1,n}^{†},\;\;{\tilde{g}}_{1,n}^{†}U_{n}], \end{array}$$
  where ${\tilde{g}}_{1,n}^{†}=g_{1}^{†}{\bar{U}}_{n}$. The slow-fading user by definition knows $g_{1}$, so it can decode ${\bar{U}}_{n}$ which yields $2\frac{T^{\prime }}{T}$ degrees of freedom. The remaining $\frac{T^{\prime }}{T}\left(T-2\right)$ observations in ${\tilde{g}}_{1,n}^{†}U_{n}$ involve $2\frac{T^{\prime }}{T}\left(T-2\right)$ unknowns, so they require a further $\frac{T^{\prime }}{T}\left(T-2\right)$ independent observations for reliable decoding.
  The components of $y_{1}^{†}=\left[y_{1,1}^{†},\;\;\cdots ,\;\;y_{1,\ell }^{†}\right]$ are
  (47) $$\begin{array}{cc} y_{1,n}^{†}= & [h_{1,n}^{†}{\bar{U}}_{n},\;\;h_{1,n}^{†}{\bar{U}}_{n}U_{n}],\;n=1,\;\ldots ,\;\ell \\ = & [{\tilde{h}}_{1,n}^{†},\;\;{\tilde{h}}_{1,n}^{†}U_{n}], \end{array}$$
  where ${\tilde{h}}_{1,n}^{†}=h_{1,n}^{†}{\bar{U}}_{n}$ is the equivalent channel estimated by the fast-fading user. The fast-fading user saves ${\tilde{h}}_{1,n}^{†}U_{n}$ for interference cancellation in the upcoming steps.
2. The transmitter sends a second super symbol intended for the fast-fading user:
  (48) $$X_{2}=\left[X_{2,1},\;\;\cdots ,\;\;X_{2,\ell }\right],$$
  where
  (49) $$X_{2,n}=\left[{\tilde{U}}_{n},\;\;{\tilde{U}}_{n}V_{n}\right],\;n=1,\;\ldots ,\;\ell ,$$
  ${\tilde{U}}_{n}\in C^{2\times 2}$ is diagonal and includes 2 independent symbols intended for the slow-fading user, and $V_{n}\in C^{2\times (T-2)}$ contains independent symbols intended for the fast-fading user. The components of $y_{2}^{†}=\left[y_{2,1}^{†},\;\;\cdots ,\;\;y_{2,\ell }^{†}\right]$ are
  (50) $$\begin{array}{cc} y_{2,n}^{†}= & [h_{2,n}^{†}{\tilde{U}}_{n},\;\;h_{2,n}^{†}{\tilde{U}}_{n}V_{n}],\;n=1,\;\ldots ,\;\ell \\ = & [{\tilde{h}}_{2,n}^{†},\;\;{\tilde{h}}_{2,n}^{†}V_{n}], \end{array}$$
  where ${\tilde{h}}_{2,n}^{†}=h_{2,n}^{†}{\tilde{U}}_{n}$ is the equivalent channel estimated by the fast-fading user. The fast-fading user saves ${\tilde{h}}_{2,n}^{†}V_{n}$, which includes $\frac{T^{\prime }}{T}\left(T-2\right)$ independent observations about $2\frac{T^{\prime }}{T}\left(T-2\right)$ unknowns, and hence, an additional $\frac{T^{\prime }}{T}\left(T-2\right)$ observations are needed to decode $V_{n}$. The components of $y_{2}^{{}^{\prime }†}=\left[y_{2,1}^{{}^{\prime }†},\;\;\cdots ,\;\;y_{2,\ell }^{{}^{\prime }†}\right]$ are
  (51) $$\begin{array}{cc} y_{2,n}^{\prime }= & [g_{2}^{†}{\tilde{U}}_{n},\;\;g_{2}^{†}{\tilde{U}}_{n}V_{n}],\;n=1,\;\ldots ,\;\ell \\ = & [{\tilde{g}}_{2,n}^{†},\;\;{\tilde{g}}_{2,n}^{†}V_{n}], \end{array}$$
  where ${\tilde{g}}_{2,n}^{†}=g_{2}^{†}{\tilde{U}}_{n}$ is the equivalent channel estimated by the slow-fading user; the slow-fading user saves ${\tilde{g}}_{2,n}^{†}V_{n}$ for the upcoming steps. Knowing $g_{2}$, the slow-fading user achieves $2\frac{T^{\prime }}{T}$ further degrees of freedom from decoding ${\tilde{U}}_{n}$.
3. The transmitter emits a third super symbol consisting of a linear combination of the signals generated from the first and the second super symbols.
  (52) $$X_{3}=\left[X_{3,1},\;\;\cdots ,\;\;X_{3,\ell }\right],$$
  where
  (53) $$X_{3,n}=\left[{\hat{U}}_{n},\;\;{\hat{U}}_{n}\left({\tilde{h}}_{1,n}^{†}U_{n}+{\tilde{g}}_{2,n}^{†}V_{n}\right)\right],\;n=1,\;\ldots ,\;\ell ,$$
  ${\hat{U}}_{n}\in C^{2\times 2}$ is diagonal and contains 2 independent symbols intended for the slow-fading user, and hence, the slow-fading user achieves further $2\frac{T^{\prime }}{T}$ degrees of freedom.
  The slow-fading user cancels ${\tilde{g}}_{2,n}^{†}V_{n}$ saved during the second super symbol and obtains ${\tilde{h}}_{1,n}^{†}U_{n}$, which includes the additional independent $\frac{T^{\prime }}{T}\left(T-2\right)$ observations needed for decoding $U_{n}$. Therefore, the slow-fading user achieves $2\frac{T^{\prime }}{T}\left(T-2\right)$ further degrees of freedom. The fast-fading user estimates the equivalent channel ${\tilde{h}}_{3,n}^{†}=h_{3,n}^{†}{\hat{U}}_{n}$, cancels ${\tilde{h}}_{1,n}^{†}U_{n}$ saved during the first super symbol, and obtains ${\tilde{g}}_{2,n}^{†}V_{n}$ which contains the additional observations needed for decoding $V_{n}$. Hence, the fast-fading user achieves $2\frac{T^{\prime }}{T}\left(T-2\right)$ degrees of freedom.

In aggregate, over $3T^{\prime }$ time instants, the slow-fading and fast-fading user achieve the degrees of freedom

(54) $$d^{\prime }=6\frac{T^{\prime }}{T}+2\frac{T^{\prime }}{T}\left(T-2\right),\;d=2\frac{T^{\prime }}{T}\left(T-2\right).$$

This completes the proof of Theorem 6. □

**Theorem** **7.** *An outer bound on the degrees of freedom region of the fading broadcast channel characterized by Equation (1), with one slow-fading and one fast-fading user having delayed CSIT, is*

(55) $$\begin{array}{cc} \frac{d^{\prime }}{2}+d & \leq 1, \end{array}$$

(56) $$\begin{array}{cc} d^{\prime }+\frac{d}{2} & \leq 1, \end{array}$$

(57) $$\begin{array}{cc} d & \leq 1-\frac{1}{T}. \end{array}$$

**Proof.**  The inequality (57) represents the single-user outer bound [21]. We prove the bound (55) as follows. We enhance the original channel by giving both users global CSIR. In addition, the channel output of the fast-fading user, y(t), is given to the slow-fading user. Therefore, the channel outputs at time instant *t* are $\left(y^{\prime }\left(t\right),y\left(t\right),H\right)$ at the slow-fading user, and (y(t),H) at the fast-fading user. The enhanced channel is physically degraded [26,27], hence, removing the delayed CSIT does not reduce the capacity [28]. Additionally,

(58) $$\begin{array}{cc} R^{\prime }\leq & I\left(x\left(t\right);y^{\prime }\left(t\right),y\left(t\right)|U,H\right)=h\left(y^{\prime }\left(t\right),y\left(t\right)|U,H\right)-h\left(y^{\prime }\left(t\right),y\left(t\right)|U,x\left(t\right),H\right)\\ R\leq & I(U;y(t)|H)=h(y(t)|H)-h(y(t)|U,H), \end{array}$$

where *U* is an auxiliary random variable, and $U\to x\to (y^{\prime }\left(t\right),y\left(t\right))$ forms a Markov chain. Therefore,

(59) $$\begin{array}{cc} \frac{R^{\prime }}{2}+R\leq & h\left(y\left(t\right)|H\right)+\frac{1}{2}h\left(y^{\prime }\left(t\right),y\left(t\right)|U,H\right)-h\left(y\left(t\right)|U,H\right)+o\left(\log\left(\rho \right)\right)\\ \leq & \log\left(\rho \right)+\frac{1}{2}h\left(y^{\prime }\left(t\right),y\left(t\right)|U,H\right)-h\left(y\left(t\right)|U,H\right)+o\left(\log\left(\rho \right)\right) \end{array}$$

(60) $$\begin{array}{cc} \leq & \log\left(\rho \right)+\max_{\mathrm{Tr}\left\{\Sigma_{x}\right\}\leq \rho ,\Sigma_{x}≽0}E_{H}\left\{\frac{1}{2}\log\left|I+H\Sigma_{x}H^{†}\right|-\log\left(1+h^{†}\left(t\right)\Sigma_{x}h\left(t\right)\right)\right\}+o\left(\log\left(\rho \right)\right) \end{array}$$

(61) $$\begin{array}{cc} \leq & \log(\rho )+o(\log(\rho )), \end{array}$$

where (59) follows since h(y(t)|H)≤log(ρ)+o(log(ρ)) [29], (60) follows from extremal entropy inequality [17,18,24], and (61) follows from Lemma 1. Hence, the bound (55) is proved. A similar argument, with the role of the two users reversed, leads to the bound (56). □

**Figure 5:**
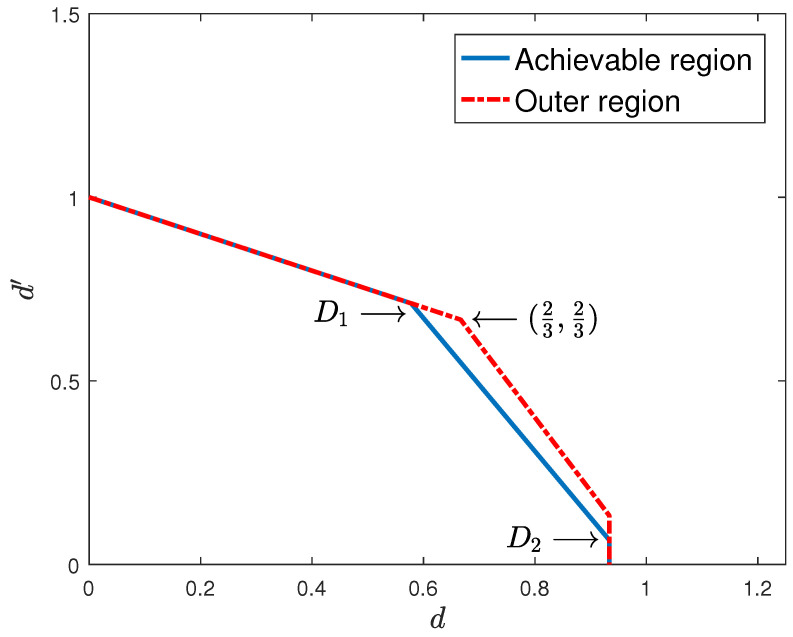
One slow-fading and one fast-fading user with delayed transmit-side channel state information (CSIT) and T=15.

**Figure 6:**
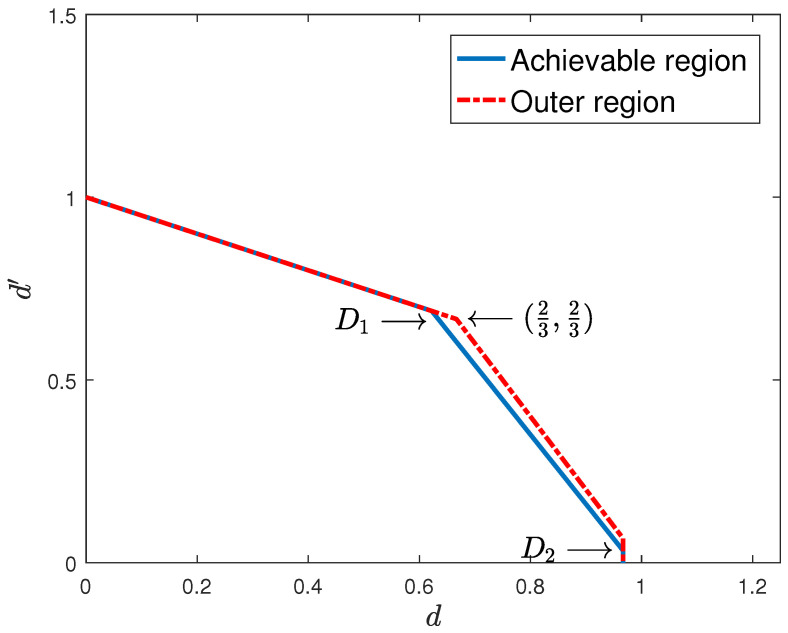
One slow-fading and one fast-fading with delayed CSIT and T=30.

**Remark** **4.** *The inner and outer bounds obtained for the two-user case partially meet, with the gap diminishing with the coherence time of the fast-fading user, as shown in Figure 5 and Figure 6 for T=15 and T=30, respectively.*

### 4.4. Transmission to Arbitrary Number of Slow-Fading and Fast-Fading Users

**Theorem** **8.** *The fading broadcast channel characterized by Equation (1), with delayed CSIT, can achieve the multiuser degrees of freedom characterized by vectors $D_{i}$,*

(62) $$\begin{array}{cc} D_{1} & :\frac{1}{1+\frac{1}{2}+\ldots +\frac{1}{m^{\prime }}}\sum_{i=1}^{m^{\prime }}e_{i}^{†}, \end{array}$$

(63) $$\begin{array}{cc} D_{2}, & \ldots ,D_{mm^{\prime }+1}:\frac{2}{3}\left(1+\frac{1}{T}\right)e_{j}^{†}+\frac{2}{3}\left(1-\frac{2}{T}\right)e_{m^{\prime }+i}^{†},\;j=1,\;\ldots ,\;m^{\prime },\;i=1,\;\ldots ,\;m, \end{array}$$

(64) $$\begin{array}{cc} D_{mm^{\prime }+2}, & \ldots ,D_{mm^{\prime }+m^{\prime }+2}:\frac{m}{T}e_{j}^{†}+\frac{1}{1+\frac{1}{2}+\ldots +\frac{1}{m}}\left(1-\frac{m}{T}\right)\sum_{i=1}^{m}e_{i}^{†},\;j=1,\;\ldots ,\;m^{\prime }, \end{array}$$

*where $e_{j}$ is the canonical coordinate vector. Their convex hull characterized an achievable degrees of freedom region.*

**Proof.**  The achievability of (62) was proved in Section 4.1 via multiuser transmission to slow-fading users. The achievability of (63) was proved in Section 4.3 via a two-user transmission to a fast-fading –slow-fading pair.

We now show the achievability of (64) via retrospective interference alignment [7] along with product superposition. Over a super symbol of length *T*, consider the following transmission:

(65) X=[U,UV],

where $U\in C^{m\times m}$ is diagonal and includes *m* independent symbols intended for the slow-fading user *j*, and $V\in C^{m\times (T-m)}$ is a super symbol containing independent symbols intended for the fast-fading users according to retrospective interference alignment [7]. Therefore, the slow-fading user decodes U. Thus, over *T* time instants, the slow-fading user achieves *m* degrees of freedom and the fast-fading users achieve $\frac{1}{1+\frac{1}{2}+\ldots +\frac{1}{m}}\left(T-m\right)$, hence, (64) is achieved. □

**Theorem** **9.** *An outer bound on the degrees of freedom of the fading broadcast channel characterized by Equation (1), with delayed CSIT, is*

(66) $$\begin{array}{cc} \sum_{j=1}^{m^{\prime }}\frac{d_{j}^{\prime }}{m^{\prime }+m}+\sum_{i=1}^{m}\frac{d_{i}}{m} & \leq 1, \end{array}$$

(67) $$\begin{array}{cc} \sum_{j=1}^{m^{\prime }}\frac{d_{j}^{\prime }}{m^{\prime }}+\sum_{i=1}^{m}\frac{d_{i}}{m^{\prime }+m} & \leq 1, \end{array}$$

(68) $$\begin{array}{cc} d_{j}^{\prime } & \leq 1,\;\forall j=1,\;\ldots ,\;m^{\prime }, \end{array}$$

(69) $$\begin{array}{cc} d_{i} & \leq 1-\frac{1}{T},\;\forall i=1,\;\ldots ,\;m. \end{array}$$

**Proof.**  The inequalities (68) and (69) represent the single-user bounds on the slow-fading and the fast-fading users, respectively [21,29]. The remainder of the proof is dedicated to establishing the bounds (66) and (67).

We enhance the channel by providing global CSIR as well as allowing full cooperation among slow-fading users and full cooperation among fast-fading users. The enhanced channel is equivalent to a broadcast channel with two users: one slow-fading equipped with $m^{\prime }$ antennas, and one fast-fading equipped with *m* antennas. Define $Y^{\prime }\in C^{m^{\prime }}$ and $Y\in C^{m}$ to be the received signals of the slow-fading and the fast-fading super-user, respectively, in the enhanced channel. We further enhance the channel by giving Y to the slow-fading user, generating a physically degraded channel since $X\to (Y^{\prime },Y)\to Y$ forms a Markov chain. Feedback including delayed CSIT has no effect on capacity [28], therefore, we remove it from consideration. Subsequently, we can utilize the Körner–Marton outer bound [19],

(70) $$\begin{array}{cc} \sum_{j=1}^{m^{\prime }}R_{j}^{\prime }\leq & I(X;Y^{\prime },Y|U,H)\\ \sum_{i=1}^{m}R_{i}\leq & I(U;Y|H). \end{array}$$

Therefore, from applying extremal entropy inequality [17,24,30] and Lemma 1,

(71) $$\begin{array}{cc} \sum_{j=1}^{m^{\prime }}\frac{R_{j}^{\prime }}{m^{\prime }+m}+\sum_{i=1}^{m}\frac{R_{i}}{m}\leq & \frac{1}{m^{\prime }+m}I\left(X;Y^{\prime },Y|U,H\right)+\frac{1}{m}I\left(U;Y|H\right)\\ = & \frac{1}{m^{\prime }+m}h\left(Y^{\prime },Y|U,H\right)+o\left(\log\left(\rho \right)\right)+\frac{1}{m}h\left(Y|H\right)-\frac{1}{m}h\left(Y|U,H\right)\\ \leq & \log(\rho )+o(\log(\rho )). \end{array}$$

Therefore, the bound (66) is proved. Similarly, we can prove the bound (67) using the same steps after switching the roles of the two users in the enhanced channel. □

## 5. Hybrid CSIT: Perfect CSIT for the Slow-Fading Users and No CSIT for the Fast-Fading Users

**Theorem** **10.** *The fading broadcast channel characterized by Equation (1), with perfect CSIT for the slow-fading users and no CSIT for the fast-fading users, can achieve the following multiuser degrees of freedom,*

(72) $$\begin{array}{cc} D_{1} & :\sum_{j=1}^{m^{\prime }}e_{j}^{†}, \end{array}$$

(73) $$\begin{array}{cc} D_{2},\;\ldots ,\;D_{m+1} & :\frac{1}{T}\sum_{j=1}^{m^{\prime }}e_{j}^{†}+\left(1-\frac{1}{T}\right)e_{i}^{†},\;i=1,\;\ldots ,\;m. \end{array}$$

*Therefore, their convex hull is also achievable.*

**Proof.**  $D_{1}$ is achieved by inverting the channels of the slow-fading users at the transmitter, then every slow-fading user achieves one degree of freedom. $D_{2},\;\ldots ,\;D_{m+1}$ in (73) are achieved using product superposition along with channel inversion as follows. The transmitted signal over *T* instants is

(74) $$X=[u,\;\;uv^{†}],$$

where $u=\sum_{j=1}^{m^{\prime }}b_{j}u_{j}$, $u_{j}$ is a symbol intended for the slow-fading user *j*, $g_{j}^{†}b_{j}=0$, and $v\in C^{T-1}$ contain independent symbols intended for the fast-fading user *i*. Each of the slow-fading users receive an interference-free signal during the first time instant of achieving one degrees of freedom. The fast-fading user estimates its equivalent channel during the first time instant and decodes v during the remaining (T−1) time instants. □

**Theorem** **11.** *An outer bound on the degrees of freedom of the fading broadcast channel characterized by Equation (1), with perfect CSIT for the slow-fading users and no CSIT for the fast-fading users, is*

(75) $$\begin{array}{cc} \sum_{j=1}^{m^{\prime }}\frac{d_{j}^{\prime }}{m^{\prime }+1}+\sum_{i=1}^{m}d_{i} & \leq 1, \end{array}$$

(76) $$\begin{array}{cc} d_{j}^{\prime } & \leq 1,\;\forall j=1,\;\ldots ,\;m^{\prime }, \end{array}$$

(77) $$\begin{array}{cc} \sum_{i=1}^{m}d_{i} & \leq 1-\frac{1}{T}. \end{array}$$

**Proof.**  The inequalities (76) represent single-user bounds for the slow-fading users [29], and (77) is a time-sharing outer bound for the fast-fading users that was established in [14,22]. It remains to prove (75), as follows.

We enhance the channel by giving global CSIR to all users and allowing full cooperation between the slow-fading users. This gives rise to an equivalent slow-fading user with $m^{\prime }$ antennas receiving $Y^{\prime }$ over an equivalent channel G and noise $Z^{\prime }$. At this point, we have a multiuser system where CSIT is available with respect to one user, but not others. We then bound the performance of this system with that of another (similar) system that has no CSIT. To do so, we use the *local statistical equivalence property* developed and used in [9,11,31]. First, we draw $\tilde{G},\tilde{Z}$ according to the distribution of $G,Z^{\prime }$ and independent of them. We enhance the channel by providing $\tilde{Y}=\tilde{G}X+\tilde{Z}$ to the slow-fading receiver and $\tilde{G}$ to all receivers. As we do *not* provide $\tilde{G}$ to the transmitter, there is no CSIT with respect to $\tilde{Y}$. According to [31], we have $h\left(\tilde{Y},Y^{\prime }|H\right)=h\left(Y^{\prime }|H\right)+o\left(\log\left(\rho \right)\right)$, where $H=(G,\tilde{G},h_{1},\;\ldots ,\;h_{m})$; therefore, we can remove $Y^{\prime }$ from the enhanced channel without reducing its degrees of freedom. This new equivalent channel has one user with $m^{\prime }$ antennas receiving $\left(\tilde{Y},H\right)$, *m* single-antenna users receiving $\left(y_{i},H\right)$, and no CSIT (In the enhanced channel after removal of $Y^{\prime }$, the transmitter and receivers still share information about G, but this random variable is now independent of all (remaining) transmit and receive variables.). Having no CSIT, the enhanced channel is in the form of a multilevel broadcast channel studied in Section 3.1, and hence, using Theorem 1,

(78) $$\begin{array}{cc} \sum_{j=1}^{m^{\prime }}R_{j}^{\prime }\leq & I\left(W;\tilde{Y}|U,H\right)+I\left(X;\tilde{Y}|U,W,H\right)\\ R_{1}\leq & I\left(U,W;y_{1}|V_{1},H\right)-I\left(W;\tilde{Y}|U,H\right)\\ R_{i}\leq & I(V_{i-1};y_{i}|V_{i},H),\;i=2,\;\ldots ,\;m. \end{array}$$

The fast-fading receiver received signals have the same distribution. By following bounding steps parallel to (22)–(24),

(79) $$\begin{array}{cc} \sum_{j=1}^{m}R_{i}\leq & \log\left(\rho \right)+o\left(\log\left(\rho \right)\right)-I\left(W;\tilde{Y}|U,H\right)-h\left(y_{1}|U,W,H\right). \end{array}$$

Therefore,

$$\begin{array}{cc} \sum_{j=1}^{m^{\prime }}\frac{R_{j}^{\prime }}{m^{\prime }+1}+\sum_{j=1}^{m}R_{i}\leq & \log\left(\rho \right)+o\left(\log\left(\rho \right)\right)+\left(\frac{1}{m^{\prime }+1}-1\right)I\left(W;\tilde{Y}|U,H\right)+\frac{h(\tilde{Y}|U,W,H)}{m^{\prime }+1} \end{array}$$

(80) $$\begin{array}{cc} \; & -h(y_{1}|U,W,H), \end{array}$$

(81) $$\begin{array}{cc} \leq & \log\left(\rho \right)+o\left(\log\left(\rho \right)\right)+\frac{h(\tilde{Y},y_{1}|U,W,H)}{m^{\prime }+1}-h\left(y_{1}|U,W,H\right) \end{array}$$

(82) $$\begin{array}{cc} \leq & \log(\rho )+o(\log(\rho )), \end{array}$$

where the last inequality follows from applying the extremal entropy inequality [17,24,30] and Lemma 1. This concludes the proof of the bound (75). □

## 6. Hybrid CSIT: Perfect CSIT for Slow-Fading Users and Delayed CSIT for Fast-Fading Users

We begin with inner and outer bounds for one slow-fading and one fast-fading user, then extend the result to multiple users. The transmitter knows the channel of the slow-fading users perfectly and instantaneously, and an outdated version of the channel of the fast-fading users.

### 6.1. Transmitting to One Slow-Fading and One Fast-Fading User

**Theorem** **12.** *For the fading broadcast channel characterized by Equation (1) with one slow-fading and one fast-fading user, with perfect CSIT for the slow-fading user and delayed CSIT for the fast-fading user, the achievable degrees of freedom region is the convex hull of the vectors*

(83) $$\begin{array}{cc} D_{1}: & \left(d^{\prime },d\right)=\left(1-\frac{1}{2T},\frac{1}{2}-\frac{1}{2T}\right), \end{array}$$

(84) $$\begin{array}{cc} D_{2}: & \left(d^{\prime },d\right)=\left(\frac{1}{T},1-\frac{1}{T}\right). \end{array}$$

**Proof.**  The degrees of freedom (84) can be achieved by product superposition, as discussed in Section 3, without CSIT. We proceed to prove the achievability of (83).

1. Consider $\left[u_{1},\;\;\cdots ,\;\;u_{T-1}\right]$ to be a complex 2×(T−1) matrix containing symbols intended for the slow-fading user, $\left[v_{1},\;\;\cdots ,\;\;v_{T-1}\right]$ intended for the fast-fading user, and b∈C is a beamforming vector so that $g^{†}b=0$. In addition, we define $u_{0}=0,v_{0}=1$. Using these components, the transmitter constructs and transmits a super symbol of length *T*, whose value at time *t* is
  (85) $$x_{1}^{†}\left(t\right)=u_{t}+b\;v_{t}.$$
  Note that $x_{1}\left(0\right)=b$ does not carry any information for either user, and serves as a pilot. The received super symbol at the slow-fading user is
  (86) $$y_{1}^{{}^{\prime }†}=\left[0,\;\;g^{†}u_{1},\;\;\cdots ,\;\;g^{†}u_{T-1}\right].$$
  The received super symbol at the fast-fading user is
  (87) $$\begin{array}{cc} y_{1}^{†}= & [h_{1}^{†}b,\;\;\left(h_{1}^{†}u_{1}+h_{1}^{†}bv_{1}\right),\;\;\cdots ,\;\;\left(h_{1}^{†}u_{T-1}+h_{1}^{†}bv_{T-1}\right)]. \end{array}$$
  The fast-fading user estimates its equivalent channel $h_{1}^{†}b$ from the received value in the first time instant. The remaining terms include symbols intended for the fast-fading user plus some interference, whose cancellation is the subject of the next step.
2. The transmitter next sends a second super symbol of length *T*,
  (88) $$x_{2}=\left[\bar{u},\;\;\bar{u}\left(h_{1}^{†}u_{1}\right),\;\;\cdots ,\;\;\bar{u}\left(h_{1}^{†}u_{T-1}\right)\right],$$
  where $\bar{u}\in C$ is a symbol intended for the slow-fading user. Hence,
  (89) $$y_{2}^{†}=\left[h_{2}\bar{u},\;\;h_{2}\bar{u}\left(h_{1}^{†}u_{1}\right),\;\;\cdots ,\;\;h_{2}\bar{u}\left(h_{1}^{†}u_{T-1}\right)\right].$$
  The fast-fading user estimates the equivalent channel $h_{2}\bar{u}$ during the first time instant and then acquires $h_{1}^{†}u_{t}$—the interference in (87). Therefore, using $y_{1},y_{2}$, the fast-fading user solves for $v_{t}$ achieving (T−1) degrees of freedom. Furthermore,
  (90) $$y_{2}^{{}^{\prime }†}=\left[g_{1}\bar{u},\;\;g_{1}\bar{u}\left(h_{1}^{†}u_{1}\right),\;\;\cdots ,\;\;g_{1}\bar{u}\left(h_{1}^{†}u_{T-1}\right)\right].$$
  The slow-fading user solves for $\bar{u}$ achieving one degree of freedom and also uses $h_{1}^{†}u_{t}$ to solve for $u_{t}$, achieving further $2\left(T-1\right)$ degrees of freedom.

In summary, during 2T instants, the slow-fading user achieves (2T−1) degrees of freedom and the fast-fading user achieves (T−1) degrees of freedom. This shows the achievability of (83). □

**Theorem** **13.** *For the fading broadcast channel characterized by Equation (1) with one slow-fading and one fast-fading user, where there is perfect CSIT for the slow-fading user and delayed CSIT for the fast-fading user, an outer bound on the degrees of freedom region is*

(91) $$\begin{array}{cc} \frac{d^{\prime }}{2}+d\leq & 1, \end{array}$$

(92) $$\begin{array}{cc} d^{\prime }\leq & 1, \end{array}$$

(93) $$\begin{array}{cc} d\leq & 1-\frac{1}{T}. \end{array}$$

**Proof.**  The inequalities (92) and (93) represent the single-user outer bounds [21,29]. It only remains to prove the outer bound (91) as follows.

1. We enhance the channel by giving global CSIR to both users and also give *y* to the slow-fading user. The enhanced channel is physically degraded, having $\left(Y^{\prime },G\right)$ at the slow-fading user and (y,G) at the fast-fading user, where $Y^{\prime }≜\left(y^{\prime },y\right)$ and G≜(h,g). In a physically degraded channel, causal feedback (including delayed CSIT) does not affect capacity [28], so we can remove the delayed CSIT with respect to the fast-fading user.
2. We now use another enhancement with the motivation to remove the remaining CSIT (noncausal, with respect to the slow-fading user). This is accomplished, similar to Theorem 11, via local statistical equivalence property [9,11,31] in the following manner. We create a channel $\tilde{G}$ and noise $\tilde{Z}$ with the same distribution but independently of the true channel and noise, and a signal $\tilde{Y}=\tilde{G}X+\tilde{Z}$. A genie will give $\tilde{Y}$ to the slow-fading receiver and $\tilde{G}$ to both receivers. It has been shown [31] that $h\left(\tilde{Y},Y^{\prime }|H\right)=h\left(Y^{\prime }|H\right)+o\left(\log\rho \right)$, where $H=(G,\tilde{G})$, therefore, we can remove $Y^{\prime }$ from the enhanced channel without reducing its degrees of freedom.
3. The enhanced channel is still physically degraded, therefore [26,27]
  (94) $$\begin{array}{cc} R^{\prime }\leq & I\left(x;\tilde{Y}|U,H\right)=h\left(\tilde{Y}|U,H\right)+o\left(\log\left(\rho \right)\right)\\ R\leq & I(U;y|H)=h(y|H)-h(y|U,H), \end{array}$$
  where *U* is an auxiliary random variable, and $U\to x\to (y^{\prime },y)$ forms a Markov chain. Therefore,
  (95) $$\begin{array}{cc} \frac{1}{2}R^{\prime }+R\leq & h\left(y|H\right)+\frac{1}{2}h\left(\tilde{Y}|U,H\right)-h\left(y|U,H\right)+o\left(\log\left(\rho \right)\right)\\ \leq & \log(\rho )+o(\log(\rho )), \end{array}$$
  where the last inequality follows from extremal entropy inequality and Lemma 1 [17,24,30]. This concludes the proof of the bound (91).

□

**Figure 7:**
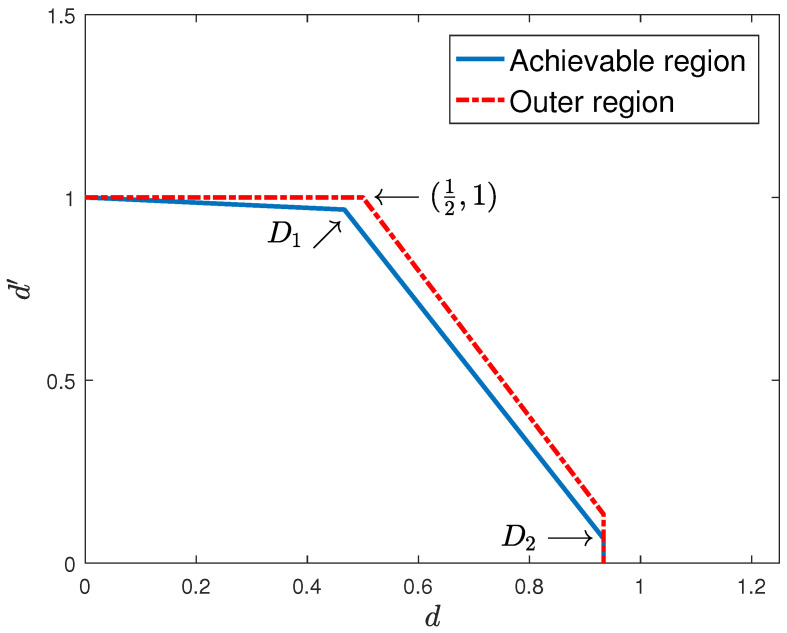
One slow-fading and one fast-fading user with hybrid CSIT and T=15.

**Figure 8:**
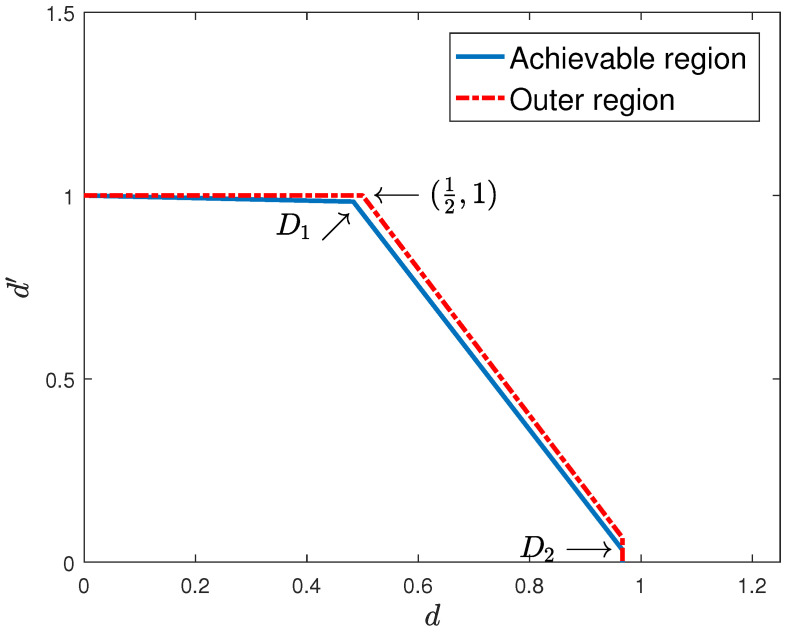
One slow-fading and one fast-fading user with hybrid CSIT and T=30.

**Remark** **5.** *For the above broadcast channel with hybrid CSIT, the achievable sum degrees of freedom is $d_{\mathrm{sum}}=\frac{3}{2}-\frac{1}{T}$, and the outer bound on the sum degrees of freedom is $d_{\mathrm{sum}}\leq \frac{3}{2}$. The gap decreases with the fast-fading user coherence time (see Figure 7 and Figure 8).*

### 6.2. Multiple Slow-Fading and Fast-Fading Users

**Theorem** **14.** *The fading broadcast channel characterized by Equation (1), with perfect CSIT for the slow-fading users and delayed CSIT for the fast-fading users, can achieve the following degrees of freedom,*

(96) $$\begin{array}{cc} D_{1} & :\sum_{j=1}^{m^{\prime }}e_{j}^{†}, \end{array}$$

(97) $$\begin{array}{cc} D_{2}, & \;\ldots ,\;D_{mm^{\prime }+1}:\left(1-\frac{1}{2T}\right)e_{j}^{†}+\left(\frac{1}{2}-\frac{1}{2T}\right)e_{i}^{†},\;j=1,\;\ldots ,\;m^{\prime },\;i=1,\;\ldots ,\;m, \end{array}$$

(98) $$\begin{array}{cc} D_{mm^{\prime }+2}, & \;\ldots ,\;D_{mm^{\prime }+m+2}:\frac{1}{T}\sum_{j=1}^{m^{\prime }}e_{j}^{†}+\left(1-\frac{1}{T}\right)e_{i}^{†},\;i=1,\;\ldots ,\;m, \end{array}$$

(99) $$\begin{array}{cc} D_{mm^{\prime }+m+3} & :\frac{m}{T}\sum_{j=1}^{m^{\prime }}e_{j}^{†}+\left(\frac{1}{1+\frac{1}{2}+\ldots +\frac{1}{m}}\left(1-\frac{m}{T}\right)\right)\sum_{i=1}^{m}e_{i}^{†}. \end{array}$$

*The achievable region consists of the convex hull of the above vectors.*

**Proof.**  $D_{1}$ is achieved by inverting the channel of the slow-fading users at the transmitter, providing one degree of freedom per slow-fading user. The achievability of $D_{2},\;\ldots ,\;D_{mm^{\prime }+1}$ was established in Section 6.1, and that of $D_{mm^{\prime }+2},\;\ldots ,\;D_{mm^{\prime }+m+2}$ was proved in Section 5 without CSIT for the fast-fading user, so it remains achievable with delayed CSIT. $D_{mm^{\prime }+m+3}$ is achieved by retrospective interference alignment [7] along with product superposition as follows. The transmitted signal over *T* instants is

(100) $$X=[\bar{U},\;\;\bar{U}V],$$

where $\bar{U}\in C^{m\times m}$ contains independent symbols intended for the slow-fading users sent by inverting the channels of the slow-fading users. Therefore, during the first *m* time instants, each slow-fading user receives an interference-free signal and achieves *m* degree of freedom; furthermore, the fast-fading users estimate their equivalent channels. During the remaining time instants, each fast-fading receiver obtains coherent observations of (T−m) transmit symbols, which are preprocessed, combined, and interference-aligned into super symbols V according to retrospective interference alignment techniques of [7]. Accordingly, each fast-fading receiver achieves $\frac{1}{1+\frac{1}{2}+\ldots +\frac{1}{m}}\left(1-\frac{m}{T}\right)$ degrees of freedom. □

**Theorem** **15.** *An outer bound on the degrees of freedom region of the fading broadcast channel characterized by Equation (1), with perfect CSIT for the slow-fading users and delayed CSIT for the fast-fading users, is*

(101) $$\begin{array}{cc} \sum_{j=1}^{m^{\prime }}\frac{d_{j}^{\prime }}{m^{\prime }+m}+\sum_{i=1}^{m}\frac{d_{i}}{m} & \leq 1, \end{array}$$

(102) $$\begin{array}{cc} \sum_{i=1}^{m}d_{i} & \leq \frac{m}{1+\frac{1}{2}+\ldots +\frac{1}{m}}, \end{array}$$

(103) $$\begin{array}{cc} d_{j}^{\prime } & \leq 1,\;j=1,\;\ldots ,\;m^{\prime }, \end{array}$$

(104) $$\begin{array}{cc} d_{i} & \leq 1-\frac{1}{T},\;i=1,\;\ldots ,\;m. \end{array}$$

**Proof.**  The inequalities (103) and (104) represent the single-user outer bounds for the slow-fading and fast-fading users, respectively [21,29]. According to Theorem 5, (102) represents an outer bound for the fast-fading users. It only remains to prove (101) as follows.

1. The original channel is enhanced by giving the users global CSIR. Furthermore, we assume full cooperation between the slow-fading users and between the fast-fading users. The resulting enhanced channel is a broadcast channel with two users: one slow-fading user equipped with $m^{\prime }$ antennas, received signal $Y^{\prime }$, channel G, and noise $Z^{\prime }$; and one fast-fading user equipped with *m* antennas, received signal Y, channel H, and noise Z.
2. We further enhance the channel by giving Y to the slow-fading user, constructing a physically degraded channel. For the enhanced channel, the slow-fading receiver is equipped with $m^{\prime }+m$ antennas and has received signal $\hat{Y}={\left[Y^{†},\;Y^{{}^{\prime }†}\right]}^{†}$, channel $\hat{G}={\left[G^{†},\;H^{†}\right]}^{†}$, and noise $\hat{Z}={\left[Z^{†},\;Z^{{}^{\prime }†}\right]}^{†}$. Since any causal feedback (including delayed CSIT) does not affect the capacity of a physically degraded channel [28], the delayed CSIT for the fast-fading receiver can be removed.
3. We now use another enhancement with the motivation to remove the remaining CSIT (noncausal, with respect to the slow-fading user). We create an artificial channel and noise—$\tilde{G}$, $\tilde{Z}$—with the same distribution but independent of $\hat{G}$, $\hat{Z}$, and a signal $\tilde{Y}=\tilde{G}X+\tilde{Z}$. A genie will give $\tilde{Y}$ to the slow-fading receiver and $\tilde{G}$ to both receivers. It has been shown [31] that $h\left(\tilde{Y},\hat{Y}|H\right)=h\left(\hat{Y}|H\right)+o\left(\log\rho \right)$, where $H=(\hat{G},\tilde{G})$, therefore, we can remove $\hat{Y}$ from the enhanced channel without reducing its degrees of freedom.
4. The enhanced channel is physically degraded without CSIT, therefore [26,27],
  (105) $$\begin{array}{cc} \sum_{j=1}^{m^{\prime }}R_{j}^{\prime } & \leq I(X;\tilde{Y}|U,H)\\ \sum_{i=1}^{m}R_{i} & \leq I(U;Y|H). \end{array}$$
  Hence,
  (106) $$\begin{array}{cc} \sum_{j=1}^{m^{\prime }}\frac{R_{j}^{\prime }}{m^{\prime }+m}+\sum_{j=1}^{m}\frac{R_{i}}{m}\leq & \frac{1}{m^{\prime }+m}h\left(\tilde{Y}|U,H\right)+\frac{1}{m}h\left(Y|H\right)-\frac{1}{m}h\left(Y|U,H\right)+o\left(\log\left(\rho \right)\right)\\ \leq & \log(\rho )+o(\log(\rho )), \end{array}$$
  where the last inequality follows from the extremal entropy inequality [17,24,30] and Lemma 1 and since h(Y|H)≤mlog(ρ)+o(log(ρ)) [29]. This concludes the proof of the bound (101).

□

## 7. Conclusions

A multiuser broadcast channel was studied where some receivers experience longer coherence intervals and have CSIR while other receivers experience a shorter coherence interval and do not have CSIR. The degrees of freedom were studied under delayed CSIT, hybrid CSIT, and no CSIT. Among the techniques employed were interference alignment and beamforming along with product superposition for the inner bounds. The outer bounds involved a bounding of the rate region of the multiuser, (discrete, memoryless,) multilevel broadcast channel. Some highlights of the results are as follows: For one slow-fading and one fast-fading user with delayed CSIT, the achievable degrees of freedom region partially meets the outer bound. For one slow-fading user with perfect CSIT and one fast-fading user with delayed CSIT, the gap between the achievable and the outer sum degrees of freedom is inversely proportional to the fast-fading user coherence time. For each of the considered CSI conditions, inner and outer bounds were also found for an arbitrary number of users.

## Figures and Tables

**Table 1:** Notation.

	Slow-Fading Users	Fast-Fading Users
number of users	$m^{\prime }$	*m*
MISO channel gains	$g_{1},\;\ldots ,\;g_{m^{\prime }}$	$h_{1},\;\ldots ,\;h_{m}$
received signals (continuous)	$y_{1}^{\prime },\;\ldots ,\;y_{m^{\prime }}^{\prime }$	$y_{1},\;\ldots ,\;y_{m}$
DMC receive variables	$Y_{1}^{\prime },\;\ldots ,\;Y_{m^{\prime }}^{\prime }$	$Y_{1},\;\ldots ,\;Y_{m}$
transmission rates	$R_{1}^{\prime },\;\ldots ,\;R_{m^{\prime }}^{\prime }$	$R_{1}\ldots ,R_{m}$
messages	$M_{1}^{\prime },\;\ldots ,\;M_{m^{\prime }}^{\prime }$	$M_{1},\;\ldots ,\;M_{m}$
degrees of freedom	$d_{1}^{\prime },\;\cdots ,\;d_{m^{\prime }}^{\prime }$	$d_{1},\;\cdots ,\;d_{m}$
coherence time	$T^{\prime }$	*T*
**General Variables**		
X	transmit signal	
ρ	signal-to-noise ratio	
$U_{i},V_{j},W$	auxiliary random variables	
H	set of all channel gains	
$D_{x}$	vertex of degrees of freedom region	
$e_{i}$	canonical coordinate vector	

## Appendix A. Proof of Theorem 1

Recall that $M_{j}^{\prime },M_{i}$ are the messages of users $j=1,\;\ldots ,\;m^{\prime }$ and i=1,…,m, respectively. We enhance the channel by assuming that user $j=1,\;\ldots ,\;m^{\prime }$ knows the messages $M_{j+1}^{\prime },\;\ldots ,\;M_{m^{\prime }}^{\prime }$ and $M_{1},\;\ldots ,\;M_{m}$ and user i=1,…,m knows the messages $M_{i+1},\;\ldots ,\;M_{m}$. Using Fano’s inequality, chain rule, and data processing inequality, we can bound the rates of the slow-fading user $j=1,\;\ldots ,\;m^{\prime }$,

(A1) $$\begin{array}{cc} nR_{j}^{\prime } & \leq I(M_{j}^{\prime };Y_{j,1}^{\prime },\;\ldots ,\;Y_{j,n}^{\prime }|M_{j+1}^{\prime },\;\ldots ,\;M_{m^{\prime }}^{\prime },M_{1},\;\ldots ,\;M_{m}) \end{array}$$

(A2) $$\begin{array}{cc} \; & =\sum_{k=1}^{n}I\left(M_{j}^{\prime };Y_{j,k}^{\prime }|U_{j,k}\right) \end{array}$$

(A3) $$\begin{array}{cc} \; & \leq \sum_{k=1}^{n}I\left(M_{j}^{\prime },U_{j,k},Y_{j-1,1}^{\prime },\;\ldots ,\;Y_{j-1,k-1}^{\prime };Y_{j,k}^{\prime }|U_{j,k}\right) \end{array}$$

(A4) $$\begin{array}{cc} \; & =\sum_{k=1}^{n}I\left(U_{j-1,k};Y_{j,k}^{\prime }|U_{j,k}\right), \end{array}$$

where

$$U_{j,k}=\left(M_{j+1}^{\prime },\;\ldots ,\;M_{m^{\prime }}^{\prime },M_{1},\;\ldots ,\;M_{m},Y_{j,1}^{\prime },\;\ldots ,\;Y_{j,k-1}^{\prime }\right),$$

$Y_{j,k}^{\prime }$ denotes the received signal of user *j* at time instant *k*,

$$U_{m^{\prime }}\to \cdots \to U_{1}\to X\to \left(Y_{1}^{\prime },\;\ldots ,\;Y_{m^{\prime }}^{\prime },Y_{1},\;\ldots ,\;Y_{m}\right)$$

forms a Markov chain, and $U_{0}=X$. The rate of slow-fading user $m^{\prime }$ can be bounded as

(A5) $$\begin{array}{cc} nR_{m^{\prime }}^{\prime }\leq & \sum_{k=1}^{n}I\left(U_{m^{\prime }-1,k};Y_{m^{\prime },k}^{\prime }|U_{m^{\prime },k}\right) \end{array}$$

(A6) $$\begin{array}{cc} = & \sum_{k=1}^{n}I\left(X_{k};Y_{m^{\prime },k}^{\prime }|U_{m^{\prime },k}\right)-\sum_{k=1}^{n}I\left(X_{k};Y_{m^{\prime },k}^{\prime }|U_{m^{\prime }-1,k}\right) \end{array}$$

(A7) $$\begin{array}{cc} \leq & \sum_{k=1}^{n}I\left(X_{k},Y_{1,k+1},\;\ldots ,\;Y_{1,n};Y_{m^{\prime },k}^{\prime }|U_{m^{\prime },k}\right)-\sum_{k=1}^{n}I\left(X_{k};Y_{m^{\prime },k}^{\prime }|U_{m^{\prime }-1,k}\right)\\ = & \sum_{k=1}^{n}I\left(Y_{1,k+1},\;\ldots ,\;Y_{1,n};Y_{m^{\prime },k}^{\prime }|U_{m^{\prime },k}\right)+\sum_{k=1}^{n}I\left(X_{k};Y_{m^{\prime },k}^{\prime }|U_{m^{\prime },k},Y_{1,k+1},\;\ldots ,\;Y_{1,n}\right) \end{array}$$

(A8) $$\begin{array}{cc} \; & -\sum_{k=1}^{n}I\left(X_{k};Y_{m^{\prime },k}^{\prime }|U_{m^{\prime }-1,k}\right) \end{array}$$

(A9) $$\begin{array}{cc} = & \sum_{k=1}^{n}I\left(W_{k};Y_{m^{\prime },k}^{\prime }|U_{m^{\prime },k}\right)+\sum_{k=1}^{n}I\left(X_{k};Y_{m^{\prime },k}^{\prime }|U_{m^{\prime },k},W_{k}\right)-\sum_{k=1}^{n}I\left(X_{k};Y_{m^{\prime },k}^{\prime }|U_{m^{\prime }-1,k}\right), \end{array}$$

where $W_{k}=Y_{1,k+1}^{n}$. Similarly,

(A10) $$\begin{array}{cc} nR_{i}\leq & I(M_{i};Y_{i,1},\;\ldots ,\;Y_{i,n}|M_{i+1},\;\ldots ,\;M_{m}) \end{array}$$

(A11) $$\begin{array}{cc} = & \sum_{k=1}^{n}I\left(M_{i};Y_{i,k}|V_{i,k}\right) \end{array}$$

(A12) $$\begin{array}{cc} = & \sum_{k=1}^{n}I\left(M_{i},V_{i,k},Y_{i-1,k+1},\;\ldots ,\;Y_{i-1,n};Y_{i,k}|V_{i,k}\right) \end{array}$$

(A13) $$\begin{array}{cc} = & \sum_{k=1}^{n}I\left(V_{i-1,k};Y_{i,k}|V_{i,k}\right), \end{array}$$

where we define $V_{i,k}≜\left(M_{i+1},\;\ldots ,\;M_{m},Y_{i,k+1},\;\ldots ,\;Y_{i,n}\right)$, which leads to the Markov chain $V_{m}\to \cdots \to V_{1}\to \left(U_{m^{\prime }},W\right)\to X\to \left(Y_{1}^{\prime },\;\ldots ,\;Y_{m^{\prime }}^{\prime },Y_{1},\;\ldots ,\;Y_{m}\right)$. Using the chain rule and Csiszár sum identity [32], we obtain the bound (A19).

(A14) $$\begin{array}{cc} R_{1}\leq & \sum_{k=1}^{n}I\left(M_{1},\;\ldots ,\;M_{m};Y_{1,k}|V_{1,k}\right) \end{array}$$

(A15) $$\begin{array}{cc} \leq & \sum_{k=1}^{n}I\left(M_{1},\;\ldots ,\;M_{m},Y_{1,k+1},\;\ldots ,\;Y_{1,n};Y_{1,k}|V_{1,k}\right) \end{array}$$

(A16) $$\begin{array}{cc} = & \sum_{k=1}^{n}I\left(M_{1},\;\ldots ,\;M_{m},Y_{1,k+1},\;\ldots ,\;Y_{1,n},Y_{m^{\prime },1}^{\prime },\;\ldots ,\;Y_{m^{\prime },k-1}^{\prime };Y_{1,k}|V_{1,k}\right) \end{array}$$

(A17) $$\begin{array}{c} -\sum_{k=1}^{n}I\left(Y_{m^{\prime },1}^{\prime },\;\ldots ,\;Y_{m^{\prime },k-1}^{\prime };Y_{1,k}|M_{1},\;\ldots ,\;M_{m},Y_{1,k+1},\;\ldots ,\;Y_{1,n}\right) \end{array}$$

(A18) $$\begin{array}{cc} = & \sum_{k=1}^{n}I\left(U_{m^{\prime },k},W_{k};Y_{1,k}|V_{1,k}\right)-\sum_{k=1}^{n}I\left(Y_{1,k+1},\;\ldots ,\;Y_{1,n};Y_{m^{\prime },k}^{\prime }|U_{m^{\prime },k}\right) \end{array}$$

(A19) $$\begin{array}{cc} = & \sum_{k=1}^{n}I\left(U_{m^{\prime },k},W_{k};Y_{1,k}|V_{1,k}\right)-\sum_{k=1}^{n}I\left(W_{k};Y_{m^{\prime },k}^{\prime }|U_{m^{\prime },k}\right). \end{array}$$

By introducing a time-sharing auxiliary random variable, *Q*, [33] and defining

(A20) $$\begin{array}{cccc} X≜ & (X_{Q},Q), & Y_{j}^{\prime } & ≜(Y_{j,Q}^{\prime },Q)\\ Y_{i}≜ & (Y_{i,Q},Q), & U_{i} & ≜(U_{i,Q},Q)\\ V_{j}≜ & (V_{j,Q},Q), & W & ≜(W_{Q},Q), \end{array}$$

we establish (7)–(10). Similarly, we can follow the same steps to prove (11)–(14) after switching the role of the two sets of variables $Y_{1}^{\prime },\;\ldots ,Y_{m^{\prime }}^{\prime }$ and $Y_{1},\;\ldots ,Y_{m}$. This completes the proof of Theorem 1.

## Appendix B. Multilevel Broadcast Channel with Degraded Message Sets

Here, we study the capacity of the multiuser, multilevel broadcast channel that is characterized by (6) with degraded message sets. In particular, $M_{0}\in \left[1:2^{nR_{0}}\right]$ is to be communicated to all receivers; and furthermore, $M_{1}\in \left[1:2^{nR_{1}}\right]$ is to be communicated to receiver $Y_{1}$ (For compactness of expression, here, we refer to each receiver by the variable denoting its received signal.). A three-receiver special case was studied by Nair and El Gamal [16], where the idea of indirect decoding was introduced, and the capacity is the set of rate pairs $\left(R_{1},R_{0}\right)$ such that

(A21) $$\begin{array}{cc} R_{0}\leq & \min\left\{I\left(U;Y_{2}\right),I\left(V;Y_{1}^{\prime }\right)\right\},\\ R_{1}\leq & I(X;Y_{1}|U),\\ R_{0}+R_{1}\leq & I\left(V;Y_{1}^{\prime }\right)+I\left(X;Y_{1}|V\right), \end{array}$$

for some pmf p(u,v)p(x|v). In the sequel, we give a generalization of Nair and El Gamal for multiuser multilevel broadcast channel.

**Theorem** **A1.**
*The capacity of multiuser multilevel broadcast channel characterized by (6), with degraded message sets, is the set of rate pairs $\left(R_{1},R_{0}\right)$ such that*

(A22) $$\begin{array}{cc} R_{0}\leq & \min\left\{I\left(U;Y_{m}\right),I\left(V;Y_{m^{\prime }}^{\prime }\right)\right\},\\ R_{1}\leq & I(X;Y_{1}|U),\\ R_{0}+R_{1}\leq & I\left(V;Y_{m^{\prime }}^{\prime }\right)+I\left(X;Y_{1}|V\right), \end{array}$$

*for some pmf p(u,v)p(x|v).*

**Proof.** The converse parallels the proof of the converse of the three-receiver case studied by Nair and El Gamal in [16] after replacing $Y_{2},Y_{1}^{\prime }$ with $Y_{m},Y_{m^{\prime }}^{\prime }$, respectively. In particular, *U* and *V* are defined as follows.

$$\begin{array}{cc} U_{k} & ≜(M_{0},Y_{1,1},\;\ldots ,\;Y_{1,k-1},Y_{m,k+1},\;\ldots ,\;Y_{m,n}),\\ V_{k} & ≜(M_{0},Y_{1,1},\;\ldots ,\;Y_{1,k-1},Y_{m^{\prime },k+1}^{\prime },\;\ldots ,\;Y_{m^{\prime },n}^{\prime }), \end{array}$$

k=1,…,n, and let *Q* be a time-sharing random variable uniformly distributed over the set {1,…,n} and independent of $X^{n},Y_{1}^{n},Y_{m,1},\;\ldots ,\;Y_{m,n},Y_{m^{\prime },1}^{\prime },\;\ldots ,\;Y_{m^{\prime },n}^{\prime }$. We then set $U=\left(U_{Q},Q\right),V=\left(V_{Q},Q\right),X=X_{Q},Y_{1}=Y_{1,Q},Y_{m}=Y_{m,Q}$, and $Y_{m^{\prime }}^{\prime }=Y_{m^{\prime },Q}^{\prime }$. This completes the converse part of the proof. The achievability part uses superposition coding and indirect decoding as follows.

- **Rate splitting:** divide the private message $M_{1}$ into two independent messages $M_{10}$ at rate $R_{10}$ and $M_{11}$ at rate $R_{11}$, where $R_{1}=R_{10}+R_{11}$.
- **Codebook generation:** fix a pmf p(u,v)p(x|v) and randomly and independently generate $2^{nR_{0}}$ sequences $u^{n}\left(m_{0}\right)$, $m_{0}\in \left[1:2^{nR_{0}}\right]$, each according to $\prod_{k=1}^{n}p_{U}\left(u_{k}\right)$. For each $m_{0}$, randomly and conditionally independently generate $2^{nR_{10}}$ sequences $v^{n}\left(m_{0},m_{10}\right)$, $m_{10}\in \left[1:2^{nR_{10}}\right]$, each according to $\prod_{k=1}^{n}p_{V|U}\left(v_{k}|u_{k}\left(m_{0}\right)\right)$. For each pair $\left(m_{0},m_{10}\right)$, randomly and conditionally independently generate $2^{nR_{11}}$ sequences $x^{n}\left(m_{0},m_{10},m_{11}\right)$, $m_{11}\in \left[1:2^{nR_{11}}\right]$, each according to $\prod_{k=1}^{n}p_{X|V}\left(x_{k}|v_{k}\left(m_{0},m_{10}\right)\right)$.
- **Encoding:** to send the message pair $\left(m_{0},m_{1}\right)=\left(m_{0},m_{10},m_{11}\right)$, the encoder transmits $x^{n}\left(m_{0},m_{10},m_{11}\right)$.
- **Decoding at the users $Y_{2},\;\ldots ,\;Y_{m}$:** decoder *i* declares that ${\hat{m}}_{0i}\in \left[1:2^{nR_{0}}\right]$ is sent if it is the unique message such that $\left(u^{n}\left({\hat{m}}_{0i}\right),y_{i}^{n}\right)\in T_{\epsilon }^{\left(n\right)}$. Hence, by law of large numbers and the packing lemma [33], the probability of error tends to zero as n→∞ if
  (A23) $$\begin{array}{cc} R_{0} & <\min_{\;2\leq i\leq m}\left\{I\left(U;Y_{i}\right)-\delta \left(\epsilon \right)\right\},\\ \; & =I\left(U;Y_{m}\right)-\delta \left(\epsilon \right), \end{array}$$
  where the last equality follows from applying data processing inequality on the Markov chain $U\to X\to Y_{1}\to Y_{2}\to \cdots \to Y_{m}$.
- **Decoding at $Y_{1}$:** decoder 1 declares that $\left({\hat{m}}_{01},{\hat{m}}_{10},{\hat{m}}_{11}\right)$ is sent if it is the unique message triple such that $\left(u^{n}\left({\hat{m}}_{01}\right),v^{n}\left({\hat{m}}_{01},{\hat{m}}_{10}\right),x^{n}\left({\hat{m}}_{01},{\hat{m}}_{10},{\hat{m}}_{11}\right),y_{1}^{n}\right)\in \left[1:2^{nR_{0}}\right]$. Hence, by law of large numbers and the packing lemma [33], the probability of error tends to zero as n→∞ if
  (A24) $$\begin{array}{cc} R_{11} & <I\left(X;Y_{1}|V\right)-\delta \left(\epsilon \right),\\ R_{10}+R_{11} & <I\left(X;Y_{1}|U\right)-\delta \left(\epsilon \right),\\ R_{0}+R_{10}+R_{11} & <I\left(X;Y_{1}\right)-\delta \left(\epsilon \right). \end{array}$$
- **Decoding at users $Y_{1}^{\prime },\;\ldots ,\;Y_{m^{\prime }}^{\prime }$:** decoder *j* decodes $m_{0}$ indirectly by declaring ${\tilde{m}}_{0j}$ is sent if it is the unique message such that $\left(u^{n}\left({\tilde{m}}_{0j}\right),v^{n}\left({\tilde{m}}_{0j},m_{10}\right),z_{j}^{n}\right)\in T_{\epsilon }^{\left(n\right)}$ for some $m_{10}\in \left[1:2^{nR_{0}}\right]$. Hence, by law of large numbers and packing lemma, the probability of error tends to zero as n→∞ if
  (A25) $$\begin{array}{cc} R_{0}+R_{10} & <\min_{\;1\leq j\leq m^{\prime }}\left\{I\left(U,V;Y_{j}^{\prime }\right)-\delta \left(\epsilon \right)\right\},\\ \; & =\min_{\;1\leq j\leq m^{\prime }}\left\{I\left(V;Y_{j}^{\prime }\right)-\delta \left(\epsilon \right)\right\},\\ \; & =I\left(V;Y_{m^{\prime }}^{\prime }\right)-\delta \left(\epsilon \right), \end{array}$$
  where the last two equalities follow from applying the chain rule and data processing inequality on the Markov chain $U\to V\to X\to Y_{1}^{\prime }\to Y_{2}^{\prime }\to \cdots \to Y_{m^{\prime }}^{\prime }$.

By combining the bounds in (A23)–(A25), substituting $R_{10}+R_{11}=R_{1}$, and eliminating $R_{10}$ and $R_{11}$ by the Fourier–Motzkin procedure [16], the proof of the achievability is completed. □
